# CD40 ligand antagonist dazodalibep in Sjögren’s disease: a randomized, double-blinded, placebo-controlled, phase 2 trial

**DOI:** 10.1038/s41591-024-03009-3

**Published:** 2024-06-05

**Authors:** E. William St. Clair, Alan N. Baer, Wan-Fai Ng, Ghaith Noaiseh, Chiara Baldini, Teresa K. Tarrant, Athena Papas, Valerie Devauchelle-Pensec, Liangwei Wang, Wenjing Xu, Tuyet-Hang Pham, Keith Sikora, William A. Rees, Ilias Alevizos

**Affiliations:** 1 Division of Rheumatology and Immunology, Duke University Department of Medicine, Durham, NC USA; 2 Department of Medicine, Johns Hopkins University School of Medicine, Baltimore, MD USA; 3 Translational and Clinical Research Institute, Newcastle University, Newcastle upon Tyne, UK; 4 NIHR Newcastle Biomedical Research Centre and NIHR Newcastle Clinical Research Facility, Newcastle upon Tyne Hospitals NHS Foundation Trust, Newcastle upon Tyne, UK; 5 HRB Clinical Research Facility, University College Cork, Cork, Ireland; 6 Division of Allergy, Clinical Immunology and Rheumatology, Department of Medicine, University of Kansas, Kansas City, KS USA; 7 Department of Clinical and Experimental Medicine, Rheumatology Unit, University of Pisa, Pisa, Italy; 8 Durham Veterans’ Administration Hospital, Durham, NC USA; 9 Division of Oral Medicine, Tufts School of Dental Medicine, Boston, MA USA; 10 Department of Rheumatology, Brest University Hospital and INSERM U1227, Brest, France; 11 Amgen Inc., Thousand Oaks, CA USA

**Keywords:** Autoimmunity, Autoimmune diseases

## Abstract

Sjögren’s disease (SjD) is a chronic, systemic autoimmune disease with no approved disease-modifying therapies. Dazodalibep (DAZ), a novel nonantibody fusion protein, is a CD40 ligand antagonist that blocks costimulatory signals between T and B cells and antigen-presenting cells, and therefore may suppress the wide spectrum of cellular and humoral responses that drive autoimmunity in SjD. This study was a phase 2, randomized, double-blinded, placebo (PBO)-controlled trial of DAZ with a crossover stage in two distinct populations of participants with SjD. Population 1 had moderate-to-severe systemic disease activity and population 2 had an unacceptable symptom burden and limited systemic organ involvement. All participants had a diagnosis of SjD, with 21.6% and 10.1% having an associated connective tissue disease (rheumatoid arthritis or systemic lupus erythematosus) in populations 1 and 2, respectively. The remaining participants would be considered as having primary Sjögren’s syndrome. The primary endpoint for population 1 (*n* = 74) was the change from baseline in the European League Against Rheumatism Sjögren’s Syndrome Disease Activity Index at day 169. The primary endpoint for population 2 (*n* = 109) was the change from baseline in the European League Against Rheumatism Sjögren’s Syndrome Patient Reported Index at day 169. The primary endpoints (least squares mean ± standard error) were achieved with statistical significance for both population 1 (DAZ, −6.3 ± 0.6; PBO, −4.1 ± 0.6; *P* = 0.0167) and population 2 (DAZ, −1.8 ± 0.2; PBO, −0.5 ± 0.2; *P* = 0.0002). DAZ was generally safe and well tolerated. Among the most frequently reported adverse events were COVID-19, diarrhea, headache, nasopharyngitis, upper respiratory tract infection, arthralgia, constipation and urinary tract infection. In summary, DAZ appears to be a potential new therapy for SjD and its efficacy implies an important role for the CD40/CD40 ligand pathway in its pathogenesis. ClinicalTrials.gov identifier: NCT04129164.

## Abstract (web summary)

In a phase 2 trial of dazodalibep, a CD40 ligand, with a crossover stage in two distinct populations of patients with Sjögren’s disease, the compound was well tolerated and led to significantly improved disease activity.

## Main

Sjögren’s disease (SjD) is a chronic, systemic autoimmune disease in which the pathophysiologic hallmarks are the production of serum autoantibodies and mononuclear cell infiltration of the lacrimal and salivary glands^1^. The predominant symptoms are due to xerostomia (oral dryness) and xerophthalmia (ocular dryness), which are often accompanied by chronic fatigue and joint pain. Extraglandular manifestations impact 30–40% of patients with SjD^2,3^ and may affect multiple organ systems, including the musculoskeletal, pulmonary, renal, nervous, dermatological, gastrointestinal, hematological, hepatobiliary and vascular systems^4–6^. SjD may occur in association with other systemic autoimmune diseases such as rheumatoid arthritis (RA) and systemic lupus erythematosus (SLE)^7,8^. Patients with SjD report a poor health-related quality of life due largely to the burden of dryness, fatigue and pain^9–11^.

The CD40/CD40 ligand (CD40L) pathway plays a prominent role in driving innate and humoral immune responses and has been implicated in the pathophysiology of a variety of autoimmune diseases^1,12–14^. CD40L is the cognate ligand for the CD40 receptor and is expressed on both hematopoietic cells and platelets^15^. The expression of both CD40L and CD40 is upregulated in the labial salivary gland tissue of patients with SjD^13^. This costimulatory pathway is essential for ectopic germinal center formation and Ig-class switching, and stimulates the production of IFNα, TNFα, IL-6 and CXCL13 (refs. ^15,16^). Salivary gland acinar and ductal epithelial cells from patients with SjD express CD40 in combination with MHC class II molecules CD80 and CD86, and are poised to present antigens and engage in crosstalk with CD40L-expressing CD4 T cells^17^. Soluble CD40L levels are elevated in the sera of patients with SjD and may further modulate the CD40/CD40L pathway^18^.

There are no approved disease-modifying therapies for SjD, and treatment of glandular manifestations is usually aimed at controlling symptoms^19–21^. Dazodalibep (DAZ), a nonantibody fusion protein that acts as a CD40L antagonist, blocks costimulatory signals between T and B cells and antigen-presenting cells, including epithelial cells^22^. The development of first-generation anti-CD40L monoclonal antibodies (mAbs) was halted due to thromboembolic complications that were later found to be mediated by the Fc region of their mAb structure^23,24^. To circumvent this problem, DAZ antigen-binding sites were engineered into a Tn3 scaffold, a non-mAb platform, which lacked an Fc region. Unlike the first-generation anti-CD40L mAbs, DAZ does not induce platelet aggregation in vitro and the clinical trials investigating DAZ therapy to date in healthy individuals and participants with RA have not revealed a thromboembolic safety signal^25,26^.

Herein we report the results of a randomized, double-blinded, placebo (PBO)-controlled, crossover trial to assess the efficacy, safety and tolerability of DAZ in two populations of participants with SjD: one with moderate-to-severe systemic disease activity and a second with an unacceptable symptom burden and limited systemic disease; the latter group has largely been excluded from recent clinical trials^27,28^. The European League Against Rheumatism (EULAR) Sjögren’s Syndrome Disease Activity Index (ESSDAI) assessed the systemic manifestations of the disease^29^ while the EULAR Sjögren’s Syndrome Patient Reported Index (ESSPRI) quantified the extent of the patient’s symptoms^30^. Our results show a significant benefit of DAZ therapy in reducing systemic disease activity and symptoms of dryness, fatigue and pain and, therefore, provide evidence of the importance of the CD40/CD40L pathway in driving both glandular and extraglandular manifestations of SjD.

## Results

### Study design

**Extended Data Fig. 1:**
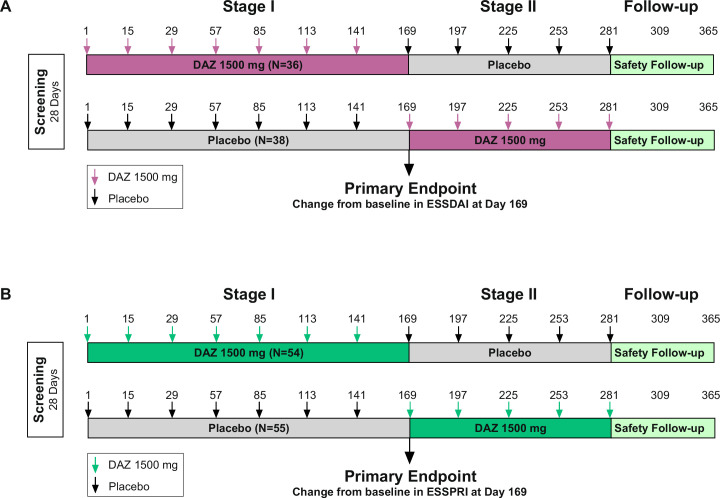
Study Design. Study design for (A) population 1 and (B) population 2. DAZ, dazodalibep; ESSDAI, EULAR Sjögren’s Syndrome Disease Activity Index; ESSPRI, EULAR Sjögren’s Syndrome Patient Reported Index.

This study was performed in adult participants with SjD who fulfilled the 2016 American College of Rheumatology/EULAR criteria. The study screened 432 participants, the first being enrolled on 14 November 2019 and the last on 28 February 2022. The experimental intervention was administered to two SjD populations. Population 1 consisted of participants with moderate-to-severe systemic disease activity (ESSDAI total score ≥5). Population 2 included participants with an unacceptable symptom burden (ESSPRI total score ≥5), a residual stimulated salivary flow of ≥0.1 ml min^−1^ and limited systemic organ involvement (ESSDAI total score <5). In stage I, PBO/DAZ 1,500 mg was administered intravenously (IV) every 2 weeks for three doses, which served as a loading dose, followed by PBO/DAZ 1,500 mg every 4 weeks for an additional four doses. A crossover design was featured to further evaluate the clinical efficacy and biological impact of DAZ. In stage II, starting at day 169, the treatment assignment was switched for participants completing stage I; participants randomized to PBO received DAZ and those randomized to DAZ received PBO in a blinded fashion every 4 weeks for five doses (Extended Data Fig. 1).

The primary endpoints for populations 1 and 2 were the change from baseline in ESSDAI total score and ESSPRI total score, respectively, at day 169. The secondary endpoints specific for population 1 included the change from baseline in ESSPRI total score at day 169 and the proportion of participants achieving ESSDAI (3) and ESSDAI (4) response (decrease of at least three or four points, respectively, from baseline in ESSDAI at day 169). The secondary endpoint specific for population 2 included the proportion of participants achieving an ESSPRI response (one or more points or 15% reduction from baseline in ESSPRI score at day 169). Secondary endpoints for both populations evaluated at day 169 included the change from baseline in Functional Assessment of Chronic Illness Therapy–Fatigue (FACIT–Fatigue) score, change from baseline in Ocular Surface Disease Index (OSDI) and change from baseline in Patient’s Global Impression of Severity (PGIS). Exploratory endpoints included the change from baseline in total stimulated salivary flow, CXCL13 levels and rheumatoid factor (RF) autoantibodies.

### Demographics and baseline disease characteristics

#### Participants with moderate-to-severe systemic disease activity

**Fig. 1:**
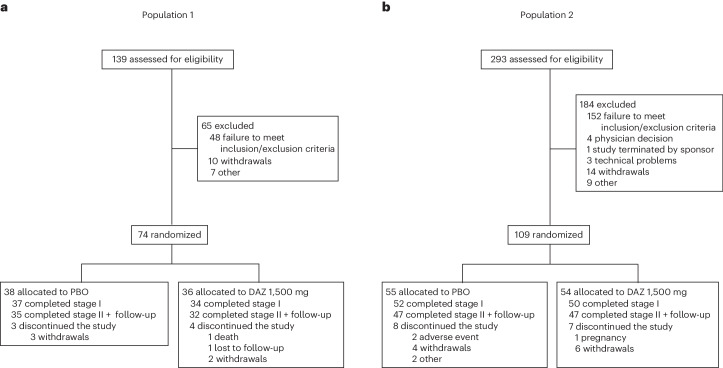
Participant disposition. Diagram of the flow of participants through the screening, enrollment, allocation, treatment and follow-up phases of the trial for population 1 (**a**) and population 2 (**b**).

Of the 139 participants screened for population 1, 74 were randomized into the two treatment groups (DAZ, *n* = 36; PBO, *n* = 38), with 71 (95.9%) completing stage I and 67 (90.5%) completing stage 2 (Fig. 1). Three participants in the PBO group discontinued the study (withdrawal, *n* = 3) and four participants in the DAZ group discontinued the study (death, *n* = 1; lost to follow-up, *n* = 1; withdrawal, *n* = 2).

The demographics and baseline disease characteristics were generally balanced between DAZ and PBO groups across both populations (Table 1). In population 1 the mean age of participants was 50.2 years; the majority were female (98.6%), predominantly white (81.1%) and not Hispanic or Latino (78.4%). The mean time from initial diagnosis to randomization was 7.4 years. At baseline, participants in population 1 had an ESSDAI total score (mean ± s.d.) of 10.7 ± 4.3 and an ESSPRI total score (mean ± s.d.) of 6.6 ± 1.7. A total of 72 (97.3%), 41 (55.4%) and 31 (41.9%) participants tested positive for SS-A, SS-B and RF autoantibodies, respectively; 16 (21.6%) participants had concomitant RA or SLE. The proportion of participants in the DAZ and PBO groups taking a disease-related medication at baseline was generally balanced (Supplementary Table 1). Most participants (86.5%) were taking at least one oral or parenteral disease-related medication at baseline, with 59.5% taking an antimalarial, 41.9% a glucocorticoid and 31.1% a conventional disease-modifying antirheumatic drug (cDMARD: azathioprine, methotrexate, parenteral methotrexate sodium or mycophenolate mofetil).

**Table 1:** Demographics and baseline disease characteristics

	Population 1		Population 2	
	PBO (*n* = 38)	DAZ 1,500 mg (*n* = 36)	PBO (*n* = 55)	DAZ 1,500 mg (*n* = 54)
Demographics				
Age, years, mean (s.d.)	48.8 (12.1)	51.7 (9.6)	49.0 (12.6)	50.9 (11.7)
Weight, kg, mean (s.d.)	69.9 (16.1)	73.2 (20.5)	71.4 (16.6)	70.0 (17.2)
Sex, *n* (%)				
Male	0	1 (2.8)	5 (9.1)	1 (1.9)
Female	38 (100)	35 (97.2)	50 (90.9)	53 (98.1)
Race, *n* (%)				
American Indian or Alaskan Native	1 (2.6)	4 (11.1)	11 (20.0)	9 (16.7)
Asian	0	2 (5.6)	5 (9.1)	11 (20.4)
Black or African American	1 (2.6)	3 (8.3)	3 (5.5)	4 (7.4)
White	34 (89.5)	26 (72.2)	31 (56.4)	23 (42.6)
Other	2 (5.3)	1 (2.8)	5 (9.1)	7 (13.0)
Ethnicity, *n* (%)				
Hispanic or Latino	8 (21.1)	8 (22.2)	23 (41.8)	22 (40.7)
Not Hispanic or Latino	30 (78.9)	28 (77.8)	32 (58.2)	32 (59.3)
Baseline disease characteristics				
ESSDAI total score, mean (s.d.)	10.1 (4.1)	11.4 (4.5)	2.5 (1.6)	3.1 (1.8)
ESSDAI total score, *n* (%)				
<10 points	18 (47.4)	15 (41.7)	55 (100)	54 (100)
≥10 points	20 (52.6)	21 (58.3)	0	0
ESSPRI total score, mean (s.d.)	6.6 (1.8)	6.6 (1.6)	6.8 (1.2)	7.1 (1.6)
Dryness	6.9 (2.3)	7.3 (1.7)	7.1 (1.1)	7.5 (1.5)
Fatigue	6.8 (2.2)	7.0 (1.7)	6.9 (1.8)	7.1 (1.8)
Pain	6.1 (2.3)	5.5 (3.0)	6.3 (1.9)	6.6 (2.6)
ESSPRI total score, *n* (%)				
<7.5 points	24 (63.2)	24 (66.7)	37 (67.3)	34 (63.0)
≥7.5 points	14 (36.8)	12 (33.3)	18 (32.7)	20 (37.0)
OSDI total score	47.0 (20.9)	48.7 (25.0)	48. 6 (19.2)	48.2 (23.8)
Schirmer’s test, *n* (%)^a^				
Yes	31 (81.6)	26 (72.2)	48 (87.3)	46 (86.8)
Positive SS-A, *n* (%)	37 (97.4)	35 (97.2)	49 (89.1)	51 (94.4)
Positive SS-B, *n* (%)	23 (60.5)	18 (50.0)	26 (47.3)	25 (46.3)
Positive RF, *n* (%)	16 (42.1)	15 (41.7)	25 (45.5)	28 (51.9)
Stimulated salivary flow, mean (s.d.)	0.97 (0.85)	0.88 (0.64)	0.83 (0.69)	1.10 (1.18)
Concomitant RA or SLE, *n* (%)	9 (23.7)	7 (19.4)	7 (12.7)	4 (7.4)

^a^≤5 mm 5 min^−1^ in at least one eye.

#### Participants with unacceptable symptom burden and limited systemic organ involvement

Of the 293 participants screened for population 2, a total of 109 were randomized into the two treatment groups (DAZ, *n* = 54; PBO, *n* = 55), with 102 (93.6%) completing stage I and 94 (86.2%) completing stage 2 (Fig. 1). Eight participants in the PBO group discontinued the study (adverse event (AE), *n* = 2; withdrawal, *n* = 4; other, *n* = 2) and seven participants in the DAZ group discontinued the study (pregnancy, *n* = 1; withdrawal, *n* = 6).

In population 2 the mean age of participants was 49.9 years and the majority were female (94.5%), predominantly white (49.5%) and not Hispanic or Latino (58.7%). The mean time from initial diagnosis to randomization was 6.0 years. At baseline the ESSPRI total score was 6.9 ± 1.4 and the ESSDAI total score was 2.8 ± 1.7. A total of 100 (91.7%), 51 (46.8%) and 53 (48.6%) participants tested positive for SS-A, SS-B and RF autoantibodies, respectively. The stimulated salivary flow rate (mean ± s.d.) at baseline was 0.96 (0.97) ml min^−1^. The majority of participants (73.4%) were taking at least one oral disease-related medication at baseline, with 66.1% taking an antimalarial, 19.3% a cholinergic agonist and 8.3% a cDMARD (Supplementary Table 2). Although the proportion of participants taking an antimalarial was generally balanced between the DAZ and PBO groups, there was an imbalance between treatment groups in participants taking a cholinergic agonist or a cDMARD. A total of 6 (11.1%) DAZ-treated participants were taking a cholinergic agonist compared with 15 (27.3%) receiving PBO, and 3 (5.6%) DAZ-treated participants were taking a cDMARD compared with six (10.9%) allocated to PBO.

### Systemic disease outcomes in population 1

**Fig. 2:**
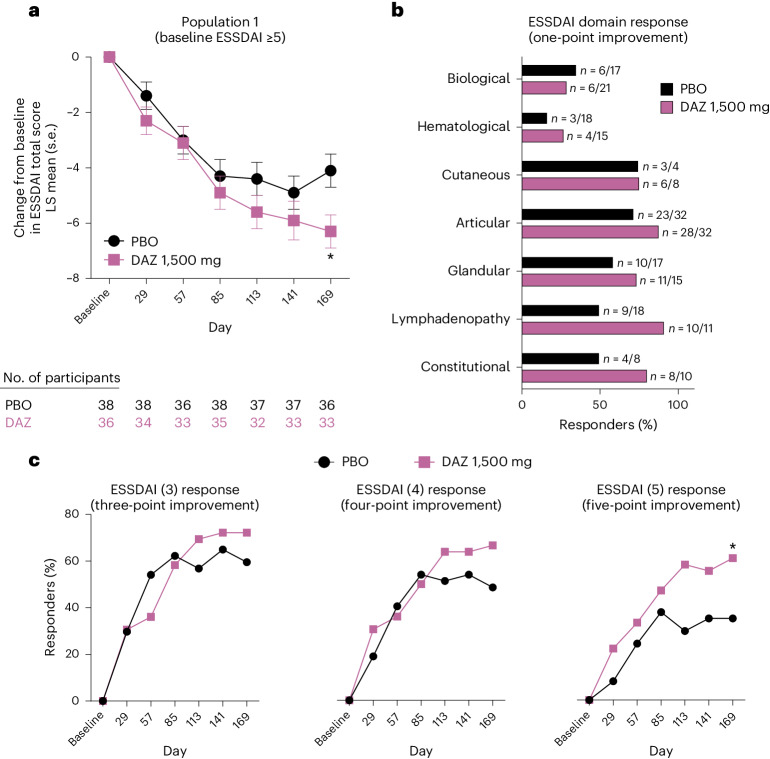
ESSDAI treatment outcomes in population 1 consisting of participants with moderate-to-severe systemic disease activity at baseline. **a**–**c**, Population 1 ESSDAI scores. **a**, Total score plotted by study visit and analyzed by MMRM (day 169, *P* = 0.0167, two-sided *t*-test). **b**, ESSDAI domain response summarized descriptively. Only participants with baseline involvement are included in the summary. **c**, ESSDAI total score responders at thresholds of three-, four- and five-point improvement from baseline (ESSDAI (5) response was a post hoc analysis) analyzed by logistic regression. *P* values were not adjusted for multiplicity; *P < 0.05. Source data

**Extended Data Fig. 2:**
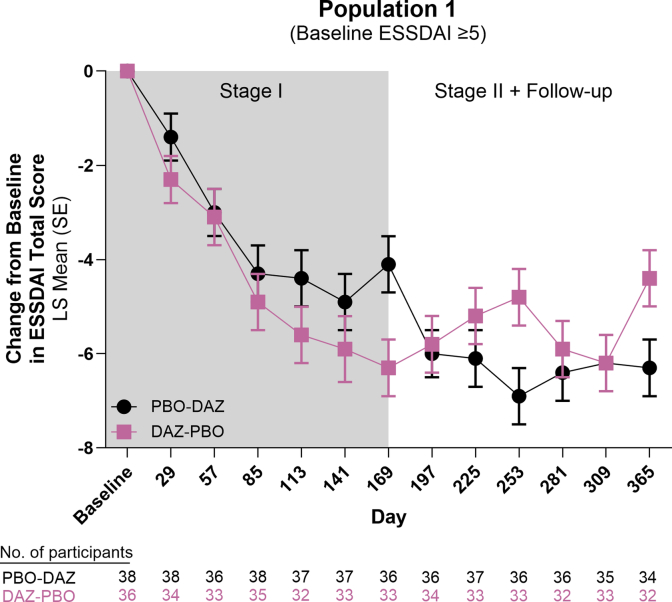
ESSDAI Total Score through Crossover Period in Population 1. The PBO-DAZ group received PBO in Stage I and transitioned to DAZ in Stage II. The DAZ-PBO group received DAZ in Stage I and transitioned to PBO in Stage II. DAZ, dazodalibep; ESSDAI, EULAR Sjögren’s Syndrome Disease Activity Index; LS, least squares; PBO, placebo; SE, standard error. Source data

The primary efficacy endpoint for population 1, the change from baseline in ESSDAI total score at day 169, was achieved with statistical significance (Fig. 2 and Table 2). The improvement in change from baseline ESSDAI total score (least squares (LS) mean ± standard error (s.e.)) at day 169 was greater in DAZ-treated participants (−6.3 ± 0.6) compared with the PBO group (−4.1 ± 0.6), yielding an LS mean difference of −2.2 ± 0.9 (*P* = 0.0167). In a subgroup analysis of population 1 we found that improvement in ESSDAI total score was similar in those participants with and without concomitant RA or SLE (Extended Data Table 1). In participants that transitioned from PBO to DAZ (PBO–DAZ) in stage II, the change from baseline in ESSDAI total score improved from −4.1 ± 0.6 at day 169 to −6.3 ± 0.6 at day 365 (Extended Data Fig. 2). In participants that switched from DAZ to PBO (DAZ–PBO) in stage II, the change from baseline in ESSDAI total score was −6.3 ± 0.6 at day 169 and decreased to −4.4 ± 0.6 at day 365.

**Table 2:** Primary and secondary efficacy endpoints

Endpoint	Population 1 Participants with moderate-to-severe systemic disease activity	
	PBO (*n* = 38)	DAZ 1,500 mg (*n* = 36)
Primary endpoint at day 169		
ESSDAI, LS mean (s.e.)	−4.1 (0.6)	−6.3 (0.6)
LS mean difference (90% CI); *P* value		−2.2 (−3.6, −0.7); 0.0167
Secondary endpoints at day 169		
ESSPRI, LS mean (s.e.)	−1.12 (0.29)	−1.80 (0.31)
LS mean difference (90% CI); *P* value		−0.68 (−1.39, 0.02); 0.1110
ESSDAI (3) responder rate, % (*n*/*N*)	59.5 (22/37)	72.2 (26/36)
Odds ratio (90% CI); *P* value		1.6 (0.7, 3.8); 0.3283
ESSDAI (4) responder rate, % (*n*/*N*)	48.6 (18/37)	66.7 (24/36)
Odds ratio (90% CI); *P* value		2.0 (0.9, 4.6); 0.1823
FACIT–Fatigue, LS mean (s.e.)	5.8 (1.6)	8.1 (1.6)
LS mean difference (90% CI); *P* value		2.3 (−1.4, 6.1); 0.3028
OSDI, LS mean (s.e.)	−14.02 (3.06)	−16.00 (3.22)
LS mean difference (90% CI); *P* value		−1.97 (−9.38, 5.44); 0.6583
PGIS	−0.5 (0.1)	−0.6 (0.1)
LS mean difference (90% CI); *P* value		−0.1 (−0.4, 0.1); 0.3629

	Population 2 Participants with unacceptable symptom burden and limited systemic organ involvement	
	PBO (*n* = 55)	DAZ 1,500 mg (*n* = 54)
Primary endpoint at day 169		
ESSPRI, LS mean (s.e.)	−0.53 (0.23)	−1.80 (0.23)
LS mean difference (90% CI); *P* value		−1.27 (−1.82, −0.73); 0.0002
Secondary endpoints at day 169		
ESSPRI responder rate, LS mean (s.e.)	32.7 (18/55)	66.7 (36/54)
Odds ratio (90% CI); *P* value		4.0 (2.0, 7.8); 0.0008
FACIT–Fatigue, LS mean (s.e.)	2.8 (1.4)	8.1 (1.4)
LS mean difference (90% CI); *P* value		5.3 (2.0, 8.7); 0.0095
OSDI, LS mean (s.e.)	−8.52 (2.94)	−13.95 (2.95)
LS mean difference (90% CI); *P* value		−5.43 (−12.31, 1.46); 0.1936
PGIS, LS mean (s.e.)	−0.4 (0.1)	−0.6 (0.1)
LS mean difference (90% CI); *P* value		−0.2 (−0.5, 0); 0.1781

Values presented as change from baseline at day 169. Continuous variables were analyzed using MMRM, and associated LS mean difference (90% confidence interval (CI)) and *P* values are reported. Binary variables were analyzed using logistic regression, and associated odds ratio (90% CI) and *P* values are reported.

A higher proportion of participants treated with DAZ achieved an ESSDAI domain response (improvement of one point or more from baseline) at day 169 in the hematological domain (26.7% (4 of 15) versus 16.7% (3 of 18)), articular (87.5% (28 of 32) versus 71.9% (23 of 32)), glandular (73.3% (11 of 15) versus 58.8% (10 of 17)), lymphadenopathy (90.9% (10 of 11) versus 50.0% (9 of 18)) and constitutional (80.0% (8 of 10) versus 50.0% (4 of 8)) domains relative to PBO (Fig. 2). An equal proportion of participants in the DAZ and PBO groups responded in the cutaneous domain (75.0% (6 of 8) versus 75.0% (3 of 4)), and a lower proportion of DAZ-treated participants had a biological domain response relative to the PBO group (28.6% (6 of 21) versus 35.3% (6 of 17)). No participants from either group had baseline involvement in the pulmonary, central nervous system and renal domains whereas one participant receiving DAZ had baseline muscular involvement (nonresponder) compared with none in the PBO group.

For the other secondary endpoints, in population 1 a higher proportion of participants in the DAZ group achieved an ESSDAI response at day 169 (three-, four- or five-point improvement from baseline) relative to the PBO group (Fig. 2). In DAZ-treated participants, 72.2% achieved an ESSDAI (3) response (versus 59.5% in the PBO group; *P* = 0.3283), 66.7% achieved an ESSDAI (4) response (versus 48.6% in the PBO group; *P* = 0.1823) and 61.1% achieved an ESSDAI (5) response (versus 35.1% in the PBO group; *P* = 0.0449; ESSDAI (5) response was a post hoc analysis).

In population 1 a numerically greater improvement at day 169 in the change from baseline ESSPRI total score was observed in DAZ-treated participants (−1.8 ± 0.3) relative to PBO (−1.1 ± 0.3, *P* = 0.1110; Table 2), a secondary endpoint in this subgroup. The individual domains of ESSPRI score also showed numerical trends of improvement from baseline to day 169 (Extended Data Table 2).

### Symptom severity outcomes in population 2

**Fig. 3:**
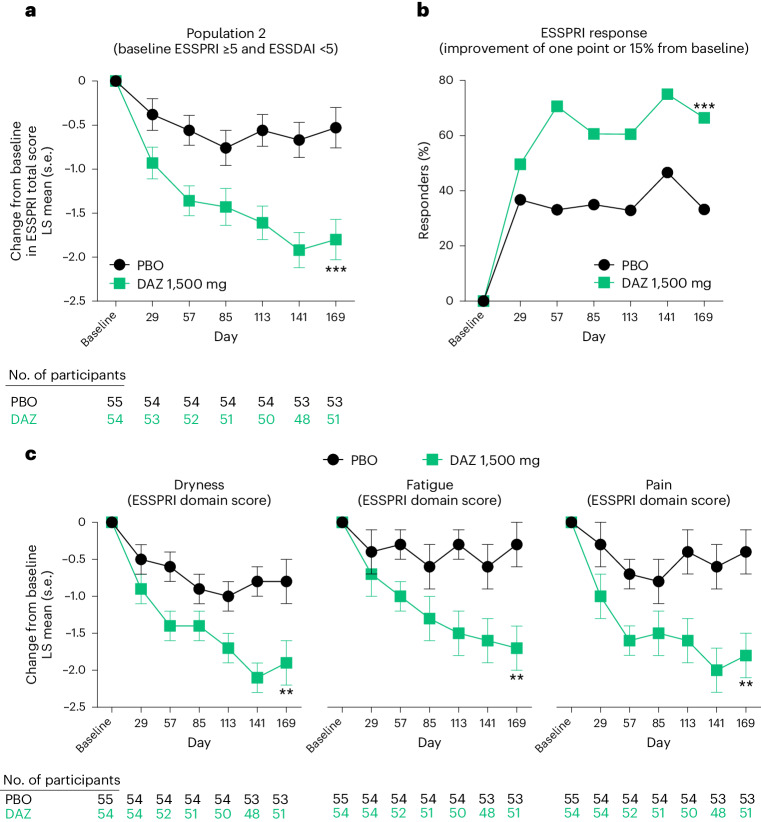
ESSPRI treatment outcomes in population 2 consisting of participants with unacceptable symptom burden and limited systemic organ involvement at baseline. **a**–**c**, Population 2 ESSPRI scores. **a**, Total ESSPRI score plotted by study visit, analyzed by MMRM (day 169, *P* = 0.0002, two-sided *t*-test). **b**, ESSPRI total score responders at a threshold of either one or more points or 15% improvement from baseline, analyzed by logistic regression (day 169, *P* = 0.0008). **c**, ESSPRI domain response for cardinal symptoms of dryness, fatigue and pain, analyzed by MMRM (day 169, dryness, *P* = 0.0066; fatigue, *P* = 0.0022; pain, *P* = 0.0010; two-sided *t*-test). *P* values were not adjusted for multiplicity; **P < 0.01, ***P < 0.001. Source data

**Extended Data Fig. 3:**
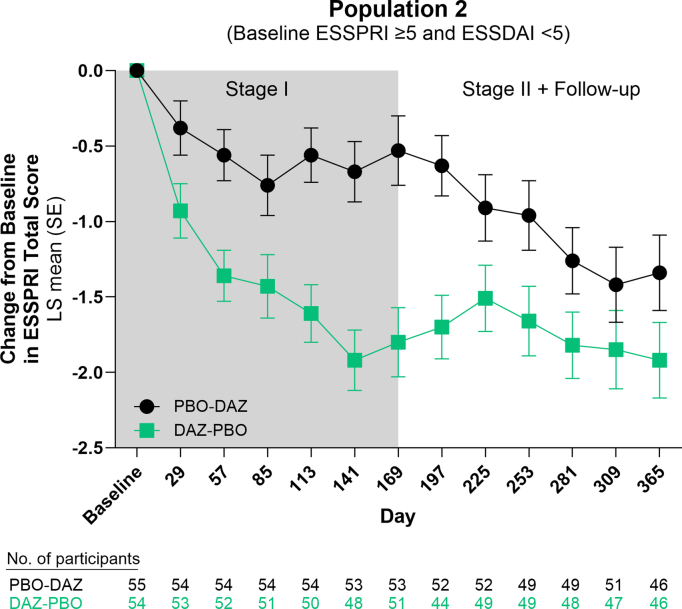
ESSPRI Total Score through Crossover Period in Population 2. The PBO-DAZ group received PBO in Stage I and transitioned to DAZ in Stage II. The DAZ-PBO group received DAZ in Stage I and transitioned to PBO in Stage II. DAZ, dazodalibep; ESSDAI, EULAR Sjögren’s Syndrome Disease Activity Index; ESSPRI, EULAR Sjögren’s Syndrome Patient Reported Index; LS, least squares; PBO, placebo; SE, standard error. Source data

The primary efficacy endpoint for population 2 was achieved with statistical significance (Fig. 3 and Table 2). In population 2 the improvement in change from baseline in ESSPRI total score (LS mean ± s.e.) at day 169 was −1.8 ± 0.2 in the DAZ group compared with −0.5 ± 0.2 in the PBO group, yielding an LS mean difference of −1.3 ± 0.3 (*P* = 0.0002). In a subgroup analysis of population 2 we found the improvement in ESSPRI total score was similar in those participants with and without RA or SLE (Extended Data Table 3). During stage II in the PBO–DAZ group, the change from baseline in ESSPRI total score improved from −0.5 ± 0.2 at day 169 to −1.3 ± 0.3 at day 365 (Extended Data Fig. 3). In the DAZ–PBO group, the change from baseline ESSPRI total score was −1.8 ± 0.2 at day 169 and −1.9 ± 0.3 at day 365.

The results favored DAZ over PBO for other secondary endpoints. A significantly higher proportion of population 2 participants in the DAZ group achieved an ESSPRI response (reduction by one or more points or ≥15% in ESSPRI total score) at day 169 relative to PBO (Fig. 3). At day 169, 66.4% of DAZ-treated participants experienced a reduction of one or more points or ≥15% in ESSPRI total score compared with 33.2% treated with PBO (*P* = 0.0008).

In population 2 the improvement in each of the three domains of the ESSPRI change from baseline scores at day 169 was significantly greater in the DAZ group compared with PBO (Fig. 3). At day 169 the changes from baseline in ESSPRI domain scores (LS mean ± s.e.) of dryness, fatigue and pain were −1.9 ± 0.3, −1.7 ± 0.3 and −1.8 ± 0.3 in the DAZ group compared with −0.8 ± 0.3, −0.3 ± 0.3 and −0.4 ± 0.3 in the PBO group, respectively (dryness, *P* = 0.0066; fatigue, *P* = 0.0022; pain, *P* = 0.0010).

**Extended Data Fig. 4:**
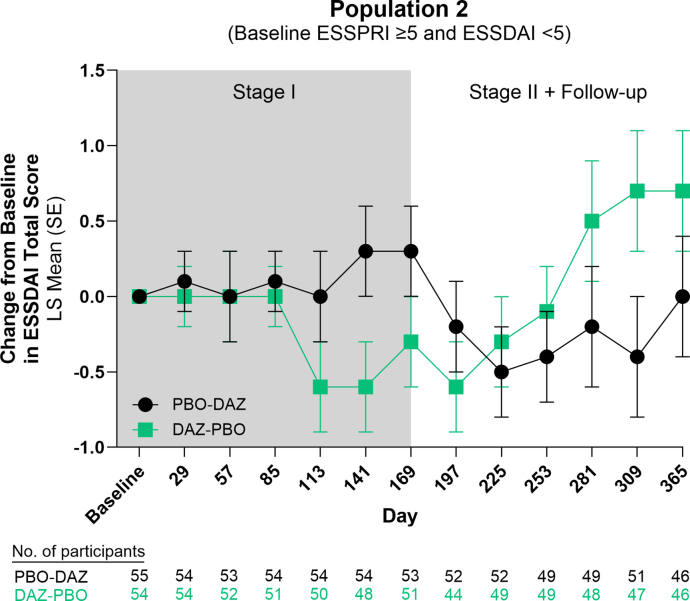
ESSDAI Total Score through Crossover Period in Population 2. The PBO-DAZ group received PBO in Stage I and transitioned to DAZ in Stage II. The DAZ-PBO group received DAZ in Stage I and transitioned to PBO in Stage II. DAZ, dazodalibep; ESSDAI, EULAR Sjögren’s Syndrome Disease Activity Index; ESSPRI, EULAR Sjögren’s Syndrome Patient Reported Index; LS, least squares; PBO, placebo; SE, standard error. Source data

In population 2 the change from baseline in ESSDAI total score at day 169 was −0.3 ± 0.3 in the DAZ group compared with 0.3 ± 0.3 in PBO (*P* = 0.2159), demonstrating a trend towards improved systemic disease manifestations with DAZ treatment (Extended Data Fig. 4). However, this group had relatively low ESSDAI total scores at baseline.

### FACIT–Fatigue, OSDI and PGIS

The observed improvements in symptoms in the DAZ group as measured by FACIT–Fatigue, OSDI and PGIS, the other secondary endpoints in this trial, were relatively consistent across both populations. In population 1, numerically greater improvement in the change from baseline FACIT–Fatigue score at day 169 was observed in the DAZ group (8.1 ± 1.6) relative to PBO (5.8 ± 1.6, *P* = 0.3028). Similarly, numerically greater improvements from baseline at day 169 were observed in the DAZ group relative to PBO in both PGIS score (DAZ, −0.6 ± 0.1; PBO, −0.5 ± 0.1, *P* = 0.3629) and OSDI score (DAZ, −16.0 ± 3.2; PBO, −14.0 ± 3.1, *P* = 0.6583).

In population 2 the DAZ group showed a statistically significant difference from PBO in the change from baseline FACIT–Fatigue score at day 169. The improvement in the change from baseline FACIT–Fatigue score was 8.1 ± 1.4 in DAZ-treated participants compared with 2.8 ± 1.4 in the PBO group (*P* = 0.0095). In population 2, numerically greater improvements at day 169 were observed in the DAZ group compared with PBO in the change from baseline in OSDI score (DAZ, −14.0 ± 3.0; PBO, −8.5 ± 2.9, *P* = 0.1936), and in change from baseline in PGIS score (DAZ, −0.6 ± 0.1; PBO, −0.4 ± 0.1, *P* = 0.1781).

**Extended Data Fig. 5:**
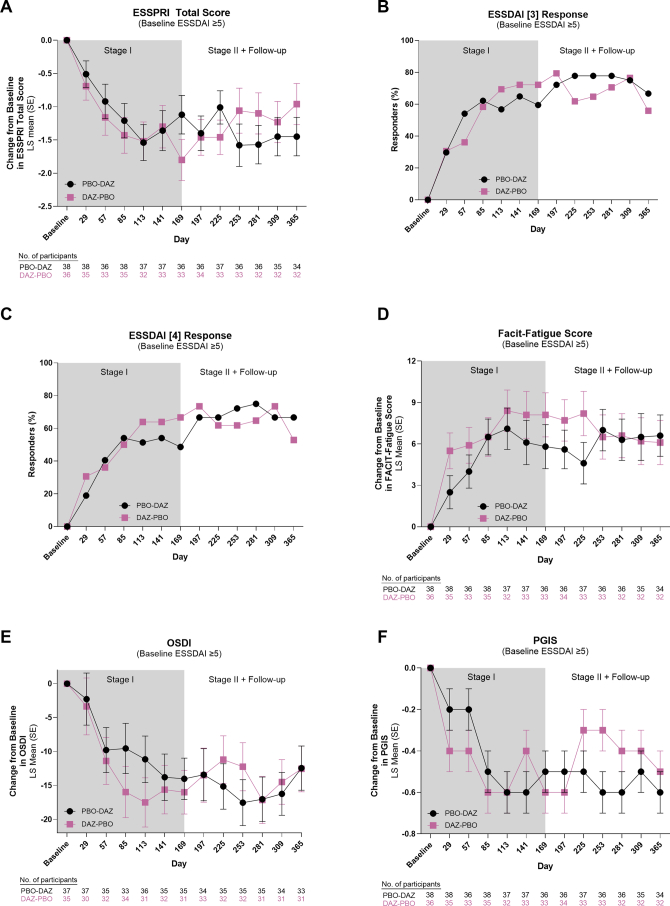
Secondary Endpoints through Crossover Period in Population 1: Participants with Moderate-to-Severe Systemic Disease Activity. Population 1 data presented by study visit for (A) ESSPRI total score, (B) ESSDAI (3) response, (C) ESSDAI (4) response, (D) FACIT-Fatigue score, (E) OSDI and (F) PGIS. The PBO-DAZ group received PBO in Stage I and transitioned to DAZ in Stage II. The DAZ-PBO group received DAZ in Stage I and transitioned to PBO in Stage II. DAZ, dazodalibep; ESSDAI, EULAR Sjögren’s Syndrome Disease Activity Index; ESSPRI, EULAR Sjögren’s Syndrome Patient Reported Index; FACIT, Functional Assessment of Chronic Illness Therapy; LS, least squares; OSDI, Ocular Surface Disease Index; PBO, placebo; PGIS, Patient Global Impression of Severity; SE, standard error. Source data

**Extended Data Fig. 6:**
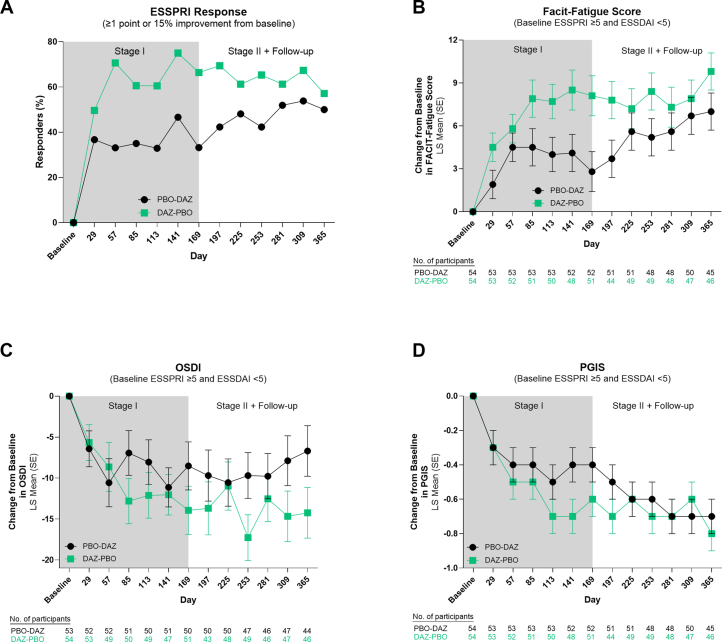
Secondary Endpoints through Crossover Period in Population 2: Participants with Unacceptable Symptom Burden and Limited Systemic Organ Involvement. Population 2 data presented by study visit for (A) ESSPRI response, (B) FACIT-Fatigue score, (C) OSDI and (D) PGIS. The PBO-DAZ group received PBO in Stage I and transitioned to DAZ in Stage II. The DAZ-PBO group received DAZ in Stage I and transitioned to PBO in Stage II. DAZ, dazodalibep; ESSDAI, EULAR Sjögren’s Syndrome Disease Activity Index; ESSPRI, EULAR Sjögren’s Syndrome Patient Reported Index; FACIT, Functional Assessment of Chronic Illness Therapy; LS, least squares; OSDI, Ocular Surface Disease Index; PBO, placebo; PGIS, Patient Global Impression of Severity; SE, standard error. Source data

The full listings of secondary efficacy endpoints assessed at day 169 in both populations are presented in Table 2, and through the crossover period in Extended Data Figs. 5 and 6.

### Safety

DAZ therapy was generally safe and well tolerated in both populations, although an imbalance in regard to COVID-19 infections was observed. There were low rates of antidrug antibodies (ADA) during stage 1 for both population 1 (2.8% ADA positive in the DAZ group versus none in PBO) and population 2 (1.9% ADA positive in the DAZ group versus none in PBO). A summary of AEs is presented in Table 3.

**Table 3:** Summary of AEs

	Stage I						Stage II + follow-up				
	Population 1			Population 2			Population 1			Population 2	
	PBO (*n* = 38)	DAZ (*n* = 36)		PBO (*n* = 55)	DAZ (*n* = 54)		PBO–DAZ (*n* = 37)	DAZ–PBO (*n* = 34)		PBO–DAZ (*n* = 52)	DAZ–PBO (*n* = 48)
≥1 AE	23 (60.5)	28 (77.8)		38 (69.1)	37 (68.5)	≥1 AE	28 (75.7)	25 (73.5)		34 (65.4)	32 (65.3)
≥1 related AE	8 (21.1)	10 (27.8)		6 (10.9)	7 (13.0)	≥1 related AE	11 (29.7)	7 (20.6)		7 (13.5)	3 (6.1)
≥1 SAE	0	1 (2.8)		1 (1.8)	3 (5.6)	≥1 SAE	1 (2.7)	3 (8.8)		1 (1.9)	2 (4.1)
≥1 AESI	0	2 (5.6)		1 (1.8)	0	≥1 AESI	0	1 (2.9)		0	2 (6.1)
Death	0	1 (2.8)		0	0	Death	0	0		0	0
≥1 AE leading to discontinuation	0	0		2 (3.6)	1 (1.9)	≥1 AE leading to discontinuation	0	1 (2.9)		1 (1.9)	0
Most frequently reported AEs (≥5% of participants in either group) Preferred term											
COVID-19	0	5 (13.9)	COVID-19	7 (12.7)	8 (14.8)	COVID-19	6 (16.2)	6 (17.6)	Nasopharyngitis	2 (3.8)	6 (12.2)
Headache	5 (13.2)	4 (11.1)	Nasopharyngitis	5 (9.1)	5 (9.3)	Upper respiratory tract infection	5 (13.5)	5 (14.7)	COVID-19	9 (17.3)	5 (10.2)
Diarrhea	0	3 (8.3)	Anemia	2 (3.6)	4 (7.4)	Diarrhea	2 (5.4)	3 (8.8)	Arthralgia	1 (1.9)	3 (6.1)
Dizziness	1 (2.6)	3 (8.3)	Diarrhea	0	3 (5.6)	Arthralgia	3 (8.1)	2 (5.9)	Constipation	0	3 (6.1)
Ligament sprain	0	3 (8.3)	Upper respiratory tract infection	4 (7.3)	2 (3.7)	Blood creatinine increased	0	2 (5.9)	Gastroesophageal reflux disease	0	3 (6.1)
Upper respiratory tract infection	2 (5.3)	3 (8.3)	Back pain	4 (7.3)	1 (1.9)	Chronic gastritis	0	2 (5.9)	Urinary tract infection	2 (3.8)	3 (6.1)
Contusion	0	2 (5.6)				Constipation	0	2 (5.9)	Headache	4 (7.7)	1 (2.0)
Device allergy	0	2 (5.6)				Enteritis	0	2 (5.9)			
Fatigue	1 (2.6)	2 (5.6)				Headache	4 (10.8)	2 (5.9)			
Hypertension	1 (2.6)	2 (5.6)				Nausea	1 (2.7)	2 (5.9)			
Nausea	3 (7.9)	2 (5.6)				Rash macular	0	2 (5.9)			
Oropharyngeal pain	0	2 (5.6)				Urinary tract infection	2 (5.4)	2 (5.9)			
Abdominal pain	2 (5.3)	0				Nasopharyngitis	3 (8.1)	0			
Back pain	2 (5.3)	0				Oral herpes	3 (8.1)	0			
Blood pressure increased	2 (5.3)	0				Sinusitis	2 (5.4)	0			
Gastroenteritis	2 (5.3)	0				Syncope	3 (8.1)	0			
Oral herpes	2 (5.3)	0				Vomiting	2 (5.4)	0			
Rhinitis	2 (5.3)	0									
Urinary tract infection	3 (7.9)	0									

Data reported as *n* (%). The PBO–DAZ group received PBO in stage I and transitioned to DAZ in stage II. The DAZ–PBO group received DAZ in stage I and transitioned to PBO in stage II.

#### Stage I

In population 1 a total of 51 participants reported an AE during stage I (DAZ, 28 (77.8%); PBO, 23 (60.5%)). The reported AEs were generally mild through to day 169 and similar in frequency between treatment groups. The most frequently reported AEs occurring in ≥5% of DAZ-treated participants, and occurring more frequently than in participants receiving PBO, were COVID-19, diarrhea, dizziness, ligament sprain, upper respiratory tract infection, contusion, device allergy, fatigue, hypertension and oropharyngeal pain. There was a single AE of special interest (AESI) of herpes zoster in a DAZ-treated participant. No participants in either group of population 1 discontinued the study during stage I due to an AE.

In population 1, two serious AEs (SAEs) were reported during stage I in a single DAZ-treated participant: this participant was a 59-year-old female who experienced COVID-19 infection and later died of an unknown cause 46 days following the last administration of DAZ (12 days after COVID-19 diagnosis). The COVID-19 infection was initially reported as an AE but was later upgraded to an SAE because the participant was considered at high risk of developing severe symptoms. The relevant medical history of this participant included congestive heart failure, hypertension, morbid obesity and pulmonary fibrosis. The SAEs occurring in population 1 during stage I were deemed by investigators to be unrelated to study medication.

In population 2, a total of 75 participants reported an AE during stage I (DAZ, 37 (68.5%); PBO, 38 (69.1%)). The most frequently reported AEs occurring in ≥5% of DAZ-treated participants, and occurring more frequently than in participants receiving PBO, were COVID-19, nasopharyngitis, anemia and diarrhea. There was a single AESI of esophageal candidiasis in a PBO-treated participant. During stage I, one participant in the DAZ group discontinued the study due to an AE (pneumonia influenza) compared with two in the PBO group (malaise and systemic lupus erythematosus).

In population 2 there were three SAEs in the DAZ group (pneumonia influenza, post-acute COVID-19 syndrome and gammopathy) and one in the PBO group (neutropenia). The participant experiencing the SAE of pneumonia influenza was an 83-year-old female, 30 days following the last administration of DAZ. The participant was hospitalized for 6 days, during which time she was diagnosed with right lower lobe pneumonia after testing positive for influenza B. The participant was later discharged from the hospital and fully recovered from the pneumonia. All participants in population 2 experiencing an SAE during stage I fully recovered from their events, and all SAEs were deemed by investigators to be unrelated to study medication.

#### Stage II

In population 1 a total of 53 participants reported an AE during stage II (DAZ–PBO, 25 (73.5%); PBO–DAZ, 28 (75.7%)). The most frequently reported AEs occurring in ≥5% participants in either group were COVID-19, upper respiratory tract infection, diarrhea, arthralgia, increased blood creatinine, chronic gastritis, constipation, enteritis, headache, nausea, rash macular, urinary tract infection, nasopharyngitis, oral herpes, sinusitis, syncope and vomiting. There was a single AESI of deep vein thrombosis (DVT) in the DAZ–PBO group. One participant in the DAZ–PBO group discontinued the study during stage II due to an AE (COVID-19) compared with none in the PBO–DAZ group.

In population 1, four SAEs were reported in three participants in the DAZ–PBO group: drug-induced liver injury (also captured as an AESI), DVT (also captured as an AESI), multiple injuries and cervical dysplasia. One SAE of chronic cholecystitis was reported in the PBO–DAZ group. The participant with a lower-extremity DVT was the same individual with drug-induced liver injury, the onset of which occurred 180 days after the final dose of DAZ. The SAEs of DVT and drug-induced liver injury were considered by investigators to be related to the study medication.

In population 2 a total of 66 participants reported an AE during stage II (DAZ–PBO, 32 (65.3%); PBO–DAZ, 34 (65.4%)). The most frequently reported AEs, occurring in ≥5% of participants in either group, were nasopharyngitis, COVID-19, arthralgia, constipation, gastroesophageal reflux disease, urinary tract infection and headache. There were three AESIs (urinary tract infection, invasive ductal breast carcinoma and herpes zoster) in the DAZ–PBO group. One participant in the PBO–DAZ group discontinued the study due to an AE (rash) compared with none in the PBO–DAZ group.

In population 2, two SAEs were reported in two participants in the DAZ–PBO group (urinary tract infection and invasive ductal breast carcinoma (both also captured as AESIs)) and one SAE was reported in the PBO–DAZ group (atrial flutter). The participant experiencing the SAE of invasive ductal breast carcinoma was a 42-year-old female, and the event was captured as both an SAE and AESI of malignant neoplasm, with onset occurring 139 days after receiving the final dose of DAZ. The SAE of invasive ductal breast carcinoma was considered by the investigators to be related to study medication.

### Total stimulated salivary flow and 28-joint assessment

**Extended Data Fig. 7:**
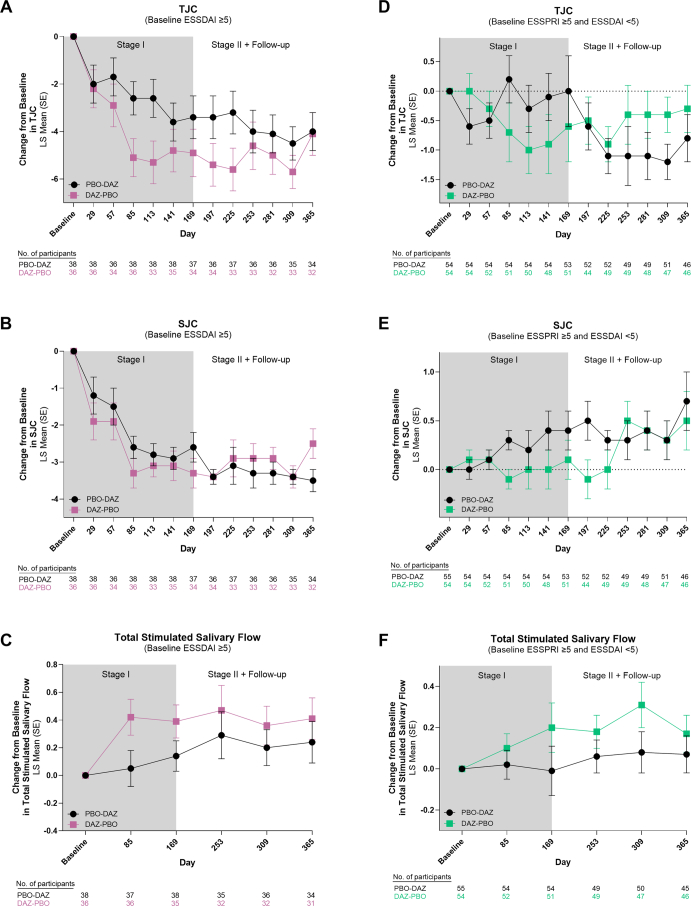
Joint Counts and Total Stimulated Salivary Flow through Crossover Period in Population 1 and Population 2. Population 1 data presented in panels A-C and Population 2 data presented in panels D-F. The PBO-DAZ group received PBO in Stage I and transitioned to DAZ in Stage II. The DAZ-PBO group received DAZ in Stage I and transitioned to PBO in Stage II. DAZ, dazodalibep; ESSDAI, EULAR Sjögren’s Syndrome Disease Activity Index; ESSPRI, EULAR Sjögren’s Syndrome Patient Reported Index; LS, least squares; PBO, placebo; SE, standard error; SJC, swollen joint count; TJC, tender joint count. Source data

In both populations, numerically greater improvements in the change from baseline total stimulated salivary flow were observed for the DAZ group relative to PBO (Extended Data Fig. 7). In population 1 the change from baseline in total stimulated salivary flow at day 169 was 0.39 ± 0.12 in DAZ-treated participants compared with 0.14 ± 0.11 in the PBO group (*P* = 0.1330). In population 2 the change from baseline in total stimulated salivary flow at day 169 was 0.20 ± 0.12 in the DAZ group compared with −0.01 ± 0.12 in the PBO group (*P* = 0.2170).

The baseline tender joint count (TJC) and swollen joint count (SJC) in population 1 were greater than those for population 2. In population 1 the baseline TJC (mean ± s.e.) in the DAZ and PBO groups was 8.6 ± 1.2 and 6.1 ± 1.0, respectively, and baseline SJC (mean ± s.e.) was 4.6 ± 0.8 and 3.6 ± 0.6, respectively. The change from baseline TJC (LS mean ± s.e.) at day 169 was −4.9 ± 1.0 in the DAZ group compared with −3.4 ± 0.9 in the PBO group (*P* = 0.2610), and the change from baseline SJC (LS mean ± s.e.) was −3.3 ± 0.4 in the DAZ group compared with −2.6 ± 0.4 in the PBO group (*P* = 0.1784; Extended Data Fig. 7). In population 2, baseline TJC and SJC were <3 and <1, respectively, in both DAZ and PBO, and were relatively unchanged during the treatment periods (Extended Data Fig. 7).

### CXCL13 and RF biomarkers

The effect of DAZ therapy on CXCL13 levels was similar for both populations (Supplementary Fig. 1). DAZ rapidly reduced CXCL13 levels during stage I, and levels remained suppressed below baseline values through to day 169. The level of CXCL13 was at its lowest between days 15 and 29. In both populations, DAZ-treated participants exhibited a significant reduction in CXCL13 relative to PBO at day 169. In population 1 the adjusted geometric mean ratio to baseline at day 169 for CXCL13 was 0.67 (0.59, 0.77) in the DAZ group compared with 1.00 (0.88, 1.14) in the PBO group (*P* = 0.0010). In population 2 the adjusted geometric mean ratio to baseline at day 169 for CXCL13 was 0.56 (0.51, 0.62) in the DAZ group compared with 1.06 (0.96, 1.17) in the PBO group (*P* < 0.0001).

In participants transitioning from PBO to DAZ in stage II, CXCL13 levels were markedly reduced after day 169 in both populations. In participants that switched from DAZ to PBO in stage II, CXCL13 recovered to near baseline levels at day 225 in population 1 and at day 253 in population 2.

At day 169, DAZ treatment significantly reduced total RF levels in both populations (Supplementary Fig. 1). In population 1 the adjusted geometric mean ratio to baseline (90% CI) at day 169 for total RF was 0.66 (0.58, 0.75) in the DAZ group compared with 1.13 (1.00, 1.29) in the PBO group (*P* < 0.0001). In population 2 the adjusted geometric mean ratio to baseline (90% CI) at day 169 for total RF was 0.54 (0.48, 0.61) in the DAZ group compared with 0.94 (0.84, 1.05) in the PBO group (*P* < 0.0001). Maximum reduction in total RF was observed at day 141 in both populations.

## Discussion

The results from our randomized, double-blinded, PBO-controlled clinical trial with a crossover stage provide evidence of the clinical efficacy of DAZ therapy for SjD and support an important role for the CD40/CD40L pathway in its pathogenesis. Among a further generation of CD40L antagonists under development, a PEGylated anti-CD40L antibody fragment has been evaluated in a phase 1, single-ascending-dose study of healthy individuals and participants with lupus and has shown predictable pharmacokinetics and acceptable tolerability without any thrombotic events^31^. In a phase 2 trial, frexalimab, an anti-CD40L humanized IgG1 monoclonal antibody lacking the FcγRIIA-activating domain, has shown promising results in participants with multiple sclerosis and is also under development for the treatment of SjD^32^. This pathway may also be inhibited by blocking the CD40 receptor. In a phase 2 study, treatment with iscalimab, a blocking and nondepleting anti-CD40 monoclonal antibody, reduced systemic disease activity in participants with SjD^33^.

CXCL13 and serum RF levels were significantly reduced by DAZ. CXCL13 is a chemokine that enables activated B and T cell migration to the germinal center, and its serum level may be reflective of germinal center formation/activity, while RF is a by-product in autoimmunity of overactive B and T cell costimulation. In a mouse model, previous studies of a T cell-dependent antigen response showed that treatment with a murine anti-CD40L Tn3 protein, similar to DAZ, led to a dose-dependent reduction in germinal center B cell frequency and complete suppression of germinal center formation^25^. In nonhuman primates, DAZ treatment suppressed T cell-dependent antibody responses to neo- and recall antigens^34^.

Most clinical trials investigating novel therapies for SjD have focused on patients with moderate-to-high systemic disease activity. While this subgroup of patients represents an unmet medical need, another important question is whether any treatment intervention can promote the recovery of glandular function and improve the symptoms of fatigue and pain. No clinical trials to date have identified any therapy with clinical efficacy for both subgroups. DAZ provided significant improvement in systemic disease activity for participants having moderate-to-severe systemic disease activity, and also led to significant improvement in the cardinal symptoms of the disease: dryness, fatigue and pain. In participants in population 1 with moderate-to-high systemic disease activity, improvement in the change from baseline in ESSDAI total score at day 169 of −6.3 ± 0.6 surpassed the three-point reduction in ESSDAI that has been defined as minimal clinically important improvement^35^. Moreover, in population 2, the subgroup with a high symptom burden, the improvement in change from baseline in ESSPRI total score at day 169 of −1.8 ± 0.2 also surpassed the one-point reduction in ESSPRI considered to be minimal clinically important improvement^35^. Analysis of multiple secondary endpoints supported the primary efficacy findings in each population.

For population 1 we did not observe significant improvement in the ESSPRI total score; however, the study was not statistically powered for this secondary endpoint. We nevertheless observed consistent trends in this subgroup favoring DAZ over PBO regarding improvement in ESSPRI total score and its individual components. Indeed, in the DAZ-treated groups the changes from baseline in ESSPRI total score at day 169 were similar for populations 1 and 2 (−1.8 (0.3) versus −1.8 (0.2), respectively). However, the changes in ESSPRI total score for the corresponding PBO groups were −1.1 (0.3) and −0.5 (0.2), respectively. Potential reasons for the numerically higher PBO response in population 1 than in population 2 include heterogeneity between the two groups owing to differences in symptom burden, levels of systemic disease activity and background medications at baseline.

Because the clinical and laboratory manifestations of RA and SLE may overlap with those of SjD, it is possible that some of the improvement in certain ESSDAI domain scores (for example, articular) for some participants with concomitant RA or SLE might not be directly attributable to changes in SjD systemic disease activity. Subgroup analysis showed that DAZ-treated participants with or without concomitant RA or SLE each had numerical improvement in their ESSDAI total scores compared with PBO. However, this comparison should be interpreted with caution due to the small sample sizes.

The safety assessment of both populations demonstrated an acceptable safety and tolerability profile of DAZ. No serious safety signals were identified in this study, although an imbalance of COVID-19 infections was noted in the DAZ group. Given the small sample size, the potential differences in epidemiologic risk and other potential confounding factors, the extent to which DAZ therapy predisposes to COVID-19-related illness remains uncertain and will be closely monitored in future studies. In stage II a participant in the DAZ–PBO group experienced an SAE of DVT; however, onset occurred 180 days after the last dose of DAZ; by contrast, in previous reports thromboembolic events associated with anti-CD40L mAbs occurred relatively soon after dosing.

There are several limitations of our phase 2 study of DAZ in SjD. Only a single DAZ dosing regimen was tested, precluding any conclusions about dose–response. The sample size of both populations was relatively small and thus increased the probability of type II error. The *P* value for significance was set at a less stringent alpha of 0.1 (two-sided) to avoid the conclusion that DAZ was ineffective in the treatment of SjD when it was actually effective. Small sample sizes are problematic in clinical trials with large PBO effects. In population 1, for example, although a large PBO response was observed for the primary endpoint, this was not unexpected because this phenomenon is commonly observed in trials of new therapies for SjD^36,37^. The small sample sizes also limited generalizability, because the study populations may not be representative of the entire population of patients with SjD. As a whole, patients with SjD and moderate-to-high systemic disease activity are a heterogeneous group with diverse extraglandular features, and the efficacy findings for population 1 may vary depending on the composition of this group. Larger studies will be needed to confirm the clinical efficacy findings, and also to extend our knowledge of safety.

DAZ is a potential new therapy for SjD, in regard to the treatment of both systemic disease activity and its unsatisfactory symptom burden. These study results support the importance of the CD40/CD40L pathway in the pathogenesis of SjD, and provide a basis for further clinical investigation of DAZ for the treatment of this systemic autoimmune disease with unmet medical need.

## Methods

### Trial design

This was a multicenter, randomized, double-blind, placebo-controlled, crossover trial to assess the efficacy, safety and tolerability of DAZ in adult participants with SjD. Participants underwent a screening period of up to 4 weeks followed by randomization and treatment for 40 weeks (24 weeks for stage I and 16 weeks for stage II). Participants were then followed for an additional 12 weeks (ClinicalTrials.gov: NCT04129164; EudraCT: 2019-002713-19).

In this study, DAZ and the saline PBO were not identical in appearance. To maintain blinding of the participants, investigators, site staff, sponsor, contract research organization and staff, a local unblinded pharmacy staff member had the responsibility of allocating, dispensing and preparing the study medication, and covering the IV bags. A separate unblinded monitor was used for the oversight of study medication management. If treatment allocation for a participant became known to the investigator or other study staff involved in the management of study participants, the sponsor was notified immediately.

All participants that participated in this study provided written informed consent. This study was approved by the Institutional Review Board/Independent Ethics Committee and conducted in accordance with ethical principles that have their origin in the Declaration of Helsinki and are consistent with both the International Council for Harmonisation/Good Clinical Practice and applicable regulatory requirements (Supplementary Table 3).

### Participants

Two populations of participants with SjD were enrolled in this study. Population 1 consisted of participants with moderate-to-severe systemic disease activity (baseline ESSDAI score ≥5). Population 2 consisted of participants with an unacceptable symptom burden and limited systemic organ involvement (baseline ESSPRI score ≥5 and baseline ESSDAI score <5).

#### Inclusion criteria

For inclusion in either population, participants were required to have a confirmed diagnosis of SjD by fulfilling the 2016 American College of Rheumatology/EULAR Classification Criteria. Participants meeting these criteria without concomitant chronic inflammatory disease (for example, RA or SLE) would be considered to have primary SjD by standard nomenclature. Participants must have been positive for either anti-Ro autoantibodies or RF, or both, at screening. Furthermore, participants must have had no history of latent or active tuberculosis before screening, with the exception of latent tuberculosis with completion of appropriate treatment.

A key criterion for inclusion in population 1 was the requirement that participants have an ESSDAI score of ≥5 at screening.

Key criteria for inclusion in population 2 were that participants (1) have an ESSPRI score ≥5 and ESSDAI score <5 at screening and (2) must have had residual salivary gland function as defined by whole stimulated salivary flow >0.1 ml min^−1^.

#### Exclusion criteria

For both populations, participants were excluded if they had a medical history of confirmed DVT or arterial thromboembolism within 2 years of screening. Furthermore, participants having risk factors for venous thromboembolism, arterial thrombosis or prothrombotic status were not permitted to enter the study. Participants requiring treatment with anticoagulant drugs were excluded, and those with concomitant polymyositis, dermatomyositis or systemic sclerosis were excluded. Participants having active malignancy or history of malignancy were not permitted to enter the study. Participants who had a positive test for, or had been treated for, hepatitis B, hepatitis C or human immunodeficiency virus infection were excluded. Moreover, participants having a history of more than one episode of herpes zoster and/or opportunistic infections within the past 12 months were not permitted to enter the study. Participants were excluded with active viral, bacterial or other infections requiring systemic treatment during the screening period, or history of more than two infections requiring IV antibiotics within 12 months before screening. Participants were also excluded if they had a positive SARS-CoV-2 test within 2 weeks before randomization, an epidemiologic risk of COVID-19 or health-related risk of developing severe COVID-19. Participants who had received live (attenuated) vaccine within the 4 weeks before screening were excluded.

In addition, for both populations, participants were excluded who had previously been treated with any biologic B cell-depleting therapy (for example, rituximab, ocrelizumab or ofatumumab) within 12 months, or with other B cell-targeting therapy (for example, belimumab) within 3 months before randomization. Participants receiving antimalarials were excluded if they had been initiated or the dose had changed within 8 weeks before screening, and participants were excluded who had any increase or initiation of new doses of cevimeline, pilocarpine or cyclosporine eye drops within 2 weeks before screening.

Key criteria warranting exclusion from population 1 included receipt of injectable corticosteroids (including intra-articular) or treatment with oral prednisone (>10 mg d^−1^) or equivalent within 6 weeks before randomization. Concomitant treatment with oral corticosteroids (≤10 mg d^−1^ prednisone or equivalent) was permitted provided the dose was stable for at least 2 weeks before the screening period. Inhaled or topical corticosteroids given for asthma, chronic obstructive pulmonary disease or dermatological conditions were permitted. Participants were excluded from population 1 that had been treated with systemic corticosteroids for indications other than Sjögren’s syndrome, RA and SLE for more than a total of 2 weeks within 24 weeks before screening.

Other key criteria warranting participant exclusion from population 1 included receiving methotrexate (MTX, >20 mg per week), azathioprine (AZA, >150 mg d^−1^), leflunomide (>20 mg d^−1^) or mycophenolate mofetil (MMF, >2 g d^−1^), or if there had been any change (including route of administration) or initiation of new dosage within 4 weeks before the screening period. Participants receiving any other DMARD, immunosuppressant or antiproliferative agent were excluded if the last dose was taken within 4 weeks before screening or within five half-lives of the drug-specific elimination period (if longer than 4 weeks).

Key criteria warranting exclusion from population 2 included the use of corticosteroids (oral, intramuscular, IV or intra-articular) within 4 weeks before the screening period. Participants were also excluded form population 2 if receiving MTX, AZA, leflunomide, another cDMARD or immunosuppressive or antiproliferative medications if the last dose was taken within 4 weeks before screening or five half-lives of the drug-specific elimination period (if longer than 4 weeks). Participants were excluded if they had had any increase or initiation of a new dose of regularly scheduled nonsteroidal anti-inflammatory drug within 2 weeks before the screening period.

### Procedures

Eligible participants were randomized 1:1 to receive three doses of either IV DAZ 1,500 mg or PBO every 2 weeks, then four additional doses every 4 weeks (stage I). Starting on day 169, participants initially randomized to DAZ received five doses of PBO every 4 weeks and those randomized to PBO received five doses of DAZ every 4 weeks; all were then followed for 12 weeks (stage II).

Randomization was stratified by ESSDAI score at screening (below 10 points versus 10 or above) for population 1, and by ESSPRI score at screening (under 7.5 points versus 7.5 or above) for population 2. To ensure balanced rates of enrollment for the two populations, a procedure was implemented where enrollment of participants in population 2 at each site was linked to that of participants in population 1 at each site, with a progressively increasing ratio of population 2 to population 1.

Rescue therapy in population 1 was defined as the initiation or increase in baseline dose of AZA, MTX, leflunomide, MMF, hydroxychloroquine or prednisone. Rescue therapy in population 2 was defined as the initiation of corticosteroids (oral, intramuscular, IV or intra-articular), MTX, AZA, leflunomide, MMF or other cDMARD, immunosuppressive or antiproliferative medication. Any initiation or increase in baseline doses of cevimeline, pilocarpine or cyclosporine eye drops was also considered a rescue therapy in population 2.

### Endpoints

The primary endpoint for population 1 was the change from baseline in ESSDAI at day 169. ESSDAI is a systemic disease activity index that includes organ-by-organ definitions of disease activity^29^. ESSDAI grades disease activity in 12 domains (cutaneous, respiratory, renal, articular, muscular, peripheral nervous system, central nervous system, hematological, glandular, constitutional, lymphadenopathic and biological). The weights of each domain were obtained by multiple regression modeling, using the Physician’s Global Assessment of Activity as gold standard. Each domain is weighted from 1 (biologic domain) to 6 (muscular domain) and has three or four levels of activity per domain, ranging from 0 (no activity) to 3 or 4 (severe activity). The theoretical range of values for ESSDAI is 0–123, with the final score being calculated as follows: final score = sum of all 12 domain scores; domain score = activity level × domain weight. Low activity status is defined as ESSDAI < 5, moderate activity as 5 ≤ ESSDAI ≤ 13 and severe activity as ESSDAI ≥ 14 (ref. ^35^). Secondary endpoints for population 1 included: the proportion of participants achieving ESSDAI (3) and ESSDAI (4) response (defined as a decrease of at least three or four points, respectively, from baseline in ESSDAI at day 169 without premature discontinuation from the study and without receiving rescue therapy); the change from baseline in FACIT–Fatigue score at day 169; the change from baseline in OSDI at day 169; and the change from baseline in PGIS at day 169. The FACIT–Fatigue scale is a participant-completed, 13-item questionnaire used to assess the impact of fatigue; its recall period is 7 days. Responses range from 0 (not at all) to 4 (very much). To calculate the total score, negatively stated items are reversed by subtracting the response from 4. Final scores are the sum of the responses, ranging 0–52. Higher scores indicate better quality of life^38^. OSDI is a 12-item questionnaire that assesses dry eye symptoms and their impact on daily activities^39^. PGIS is a single item designed to capture the participant’s perception of overall symptom severity over the previous week on a five-point categorical response scale (none, mild, moderate, severe or very severe).

The primary endpoint for population 2 was the change from baseline in ESSPRI at day 169. ESSPRI uses a numerical analog scale (ranging from 0 (no symptoms) to 10 (maximal imaginable severity)), one for the assessment of each of the three domains of dryness, fatigue and pain (articular and/or muscular). The weights of the domains are identical and the mean of the scores for the three domains represents the final score. The recall period is stated in each question as “the last 2 weeks”^30^. Secondary endpoints for population 2 included the proportion of participants achieving an ESSPRI response (defined as either one point or more or 15% reduction from baseline in ESSPRI score at day 169, without premature discontinuation from the study and without receiving rescue therapy), the change from baseline in FACIT–Fatigue score at day 169, the change from baseline in OSDI at day 169 and the change from baseline in PGIS at day 169.

Exploratory endpoints in both populations included the change from baseline in total stimulated salivary flow, change from baseline in CXCL13 and change from baseline in RF autoantibodies.

Safety assessments included the incidence of treatment-emergent AEs, treatment-emergent SAEs and treatment-emergent AESIs. If clinically relevant, changes in laboratory results, vital signs and electrocardiographic abnormalities were captured as an AE. The relatedness of an AE to study medication was provided by the site investigator, and its severity was evaluated.

### Statistical analysis

The planned sample size of 72 participants in population 1 (36 in the DAZ group and 36 in the PBO group) provided 80% power to detect a difference in mean change from baseline to day 169 in ESSDAI of 3.0 (assumed s.d. = 5) between the DAZ and PBO groups at a two-sided alpha level of 0.10 using a two-sample *t*-test.

The planned sample size of 102 participants in population 2 (51 in the DAZ group and 51 in the PBO group) provided 80% power to detect a difference in mean change from baseline to day 169 in ESSPRI of 1.0 (assumed s.d. = 2) between the DAZ and PBO groups at a two-sided alpha level of 0.10 using a two-sample *t*-test.

Because our trial was a global study with 43 sites, multiple patients were screened simultaneously across sites. Having reached the preplanned sample size of 72 and 102 participants for populations 1 and 2, respectively, a number of participants above these totals were in the process of screening and, if eligible, were allowed to be randomized and proceed to study treatment.

Efficacy analysis was based on the full analysis set, including all randomized participants who received any dose of study medication. The primary endpoint for each population was analyzed using the mixed-effect model for repeated measures (MMRM) approach. This model included fixed effects for treatment, visit, visit by treatment interaction and corresponding baseline score. Continuous secondary endpoints were analyzed using the same approach as for the primary endpoints, except that the stratification factor was included in the model.

For participants who received rescue medications before day 169, the data collected after administration of the rescue medication were not included in the analysis. The data collected after discontinuation of study treatment were included in the analysis. There was no multiplicity adjustment for the primary endpoint analysis for each population because there was only one primary comparison (DAZ 1,500 mg versus PBO) for each population. *P* < 0.1 was considered statistically significant. No multiplicity adjustment was performed for secondary endpoints.

Binary endpoints were analyzed using a logistic regression model, with treatment and corresponding baseline score included in the model. Participants who prematurely discontinued from the study were considered nonresponders for the visits after discontinuation, and participants who received rescue therapy were considered nonresponders for the visits after institution of rescue therapy.

Safety data were summarized descriptively for each population based on the safety analysis set that included all participants who received any dose of study medication.

Data analysis was performed using SAS v.9.4.

### Reporting summary

Further information on research design is available in the Nature Portfolio Reporting Summary linked to this article.

## Online content

Any methods, additional references, Nature Portfolio reporting summaries, source data, extended data, supplementary information, acknowledgements, peer review information; details of author contributions and competing interests; and statements of data and code availability are available at 10.1038/s41591-024-03009-3.

### Supplementary information

Supplementary Information Supplementary Tables 1–3 and Fig. 1.

Reporting Summary

Supplementary Data 1 Supplementary Fig. 1 source data.

### Source data

Source Data Fig. 2 Source data.

Source Data Fig. 3 Source data.

Source Data Extended Data Fig. 2 Source data.

Source Data Extended Data Fig. 3 Source data.

Source Data Extended Data Fig. 4 Source data.

Source Data Extended Data Fig. 5 Source data.

Source Data Extended Data Fig. 6 Source data.

Source Data Extended Data Fig. 7 Source data.

## Figures and Tables

**Extended Data Table 1:**
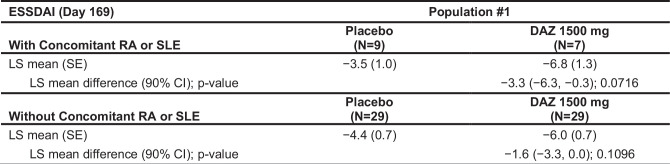
ESSDAI Total Score at Day 169 in Population 1 for Participants with and without Concomitant RA or SLE

Data values presented as change from baseline at Day 169. Data were analyzed using MMRM and associated LS mean difference (90% CI) and p-values are reported. CI, confidence interval; DAZ, dazodalibep; ESSDAI, EULAR Sjögren’s Syndrome Disease Activity Index; LS, least squares; MMRM, mixed-effect model for repeated measures; RA, rheumatoid arthritis; SE, standard error SLE, systemic lupus erythematosus.

**Extended Data Table 2:**
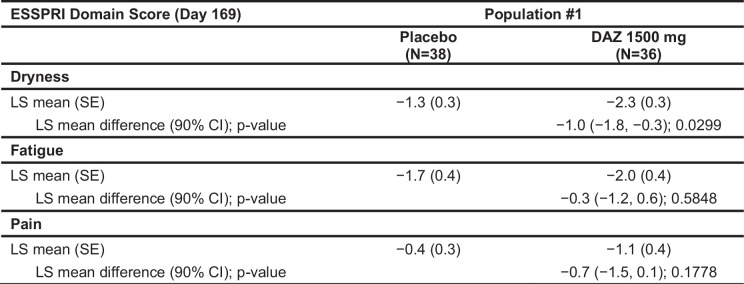
ESSPRI Domain Scores at Day 169 in Population 1

Data values presented as change from baseline at Day 169. Data analyzed using MMRM and associated LS mean difference (90% CI) and p-values are reported. CI, confidence interval; DAZ, dazodalibep; ESSPRI, EULAR Sjögren’s Syndrome Patient Reported Index; LS, least squares; SE, standard error.

**Extended Data Table 3:**
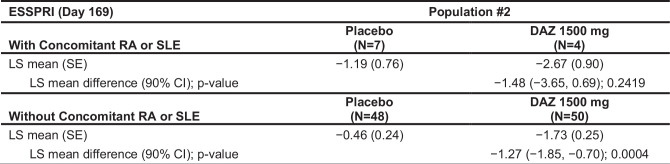
ESSPRI Total Score at Day 169 in Population 2 for Participants with and without Concomitant RA or SLE

Data values presented as change from baseline at Day 169. Data were analyzed using MMRM and associated LS mean difference (90% CI) and p-values are reported. CI, confidence interval; DAZ, dazodalibep; ESSPRI, EULAR Sjögren’s Syndrome Patient Reported Index; LS, least squares; MMRM, mixed-effect model for repeated measures; RA, rheumatoid arthritis; SE, standard error SLE, systemic lupus erythematosus.

## Data Availability

Data-sharing requests relating to data in this paper will be considered after the publication date and providing that either (1) this product and indication (or other new use) have been granted marketing authorization in both the United States and Europe or (2) clinical development discontinues and the data will not be submitted to the regulatory authorities. There is no end date for eligibility to submit a data-sharing request for these data. This may include deidentified individual patient data for variables necessary to address the specific research question in an approved data-sharing request; and also related data dictionaries, study protocol, statistical analysis plan, informed consent form and/or clinical study report. Qualified researchers may submit a request containing the research objectives, the Amgen product(s) and Amgen study/studies in scope, endpoints/outcomes of interest, statistical analysis plan, data requirements, publication plan and qualifications of the researcher(s). In general, Amgen does not grant external requests for individual patient data for the purpose of re-evaluation of safety and efficacy issues already addressed in the product labeling. A committee of internal advisors reviews requests. If not approved, requests may be further arbitrated by a Data Sharing Independent Review Panel. Requests that pose a potential conflict of interest or an actual or potential competitive risk may be declined at Amgen’s sole discretion and without further arbitration. Following approval, information necessary to address the research question will be provided under the terms of a data-sharing agreement. This may include anonymized individual patient data and/or available supporting documents, containing fragments of analysis code where provided in analysis specifications. Further details are available at the following: https://wwwext.amgen.com/science/clinical-trials/clinical-data-transparency-practices/clinical-trial-data-sharing-requestSource data are provided with this paper.
