# Supplementary material for: CD40 ligand antagonist dazodalibep in Sjögren’s disease: a randomized, double-blinded, placebo-controlled, phase 2 trial
Source: Nat Med. 2024 Jun 5;30(6):1583–92. doi: 10.1038/s41591-024-03009-3 (PMC11186761; doi:10.1038/s41591-024-03009-3)
Supplement: Supplementary file 1 — Supplementary Tables 1–3 and Fig. 1. [file 41591_2024_3009_MOESM1_ESM.pdf]

nature medicine

Supplementary information https://doi.org/10.1038/s41591-024-03009-3

CD40 ligand antagonist dazodalibep in Sjögren's disease: a randomized, double-blinded, placebo-controlled, phase 2 trial

In the format provided by the authors and unedited

Table S1. Disease-Related Medications at Baseline in Population #1

	Population #1		
	**Placebo (N=38)**	**DAZ 1500 mg (N=36)**	**Total (N=74)**
≥1 Disease-related medication at baseline, n (%)	33 (86.8)	31 (86.1)	64 (86.5)
Cholinergic agonists (systemic), n (%)	2 (5.3)	2 (5.6)	4 (5.4)
Pilocarpine	2 (5.3)	2 (5.6)	4 (5.4)
Antimalarial (systemic), n (%)	25 (65.8)	19 (52.8)	44 (59.5)
Chloroquine	0	1 (2.8)	1 (1.4)
Chloroquine phosphate	2 (5.3)	0	2 (2.7)
Hydroxychloroquine	23 (60.5)	17 (47.2)	40 (54.1)
Hydroxychloroquine sulfate	0	1 (2.8)	1 (1.4)
Glucocorticoids (local), n (%)	0	1 (2.8)	1 (1.4)
Triamcinolone	0	1 (2.8)	1 (1.4)
Glucocorticoids (systemic), n (%)	15 (39.5)	15 (41.7)	30 (40.5)
Deflazacort	1 (2.6)	1 (2.8)	2 (2.7)
Methylprednisolone	5 (13.2)	8 (22.2)	13 (17.6)
Prednisone	9 (23.7)	6 (16.7)	15 (20.3)
cDMARD (local), n (%)	1 (2.6)	1 (2.8)	2 (2.7)
Ciclosporin	1 (2.6)	1 (2.8)	2 (2.7)
cDMARD (systemic), n (%)	10 (26.3)	12 (33.3)	22 (29.7)
Azathioprine	2 (5.3)	3 (8.3)	5 (6.8)
Methotrexate	7 (18.4)	6 (16.7)	13 (17.6)
Methotrexate sodium	1 (2.6)	2 (5.6)	3 (4.1)
Mycophenolate mofetil	0	1 (2.8)	1 (1.4)

Data reported as n (%). cDMARD, conventional disease-modifying anti-rheumatic drug; DAZ, dazodalibep.

Table S2. Disease-Related Medications at Baseline in Population #2

	Population #2		
	Placebo (N=55)	DAZ 1500 mg (N=54)	Total (N=109)
≥1 Disease-related medication at baseline, n (%)	40 (72.7)	40 (74.1)	80 (73.4)
Cholinergic agonists (systemic), n (%)	13 (23.6)	6 (11.1)	19 (17.4)
Cevimeline	2 (3.6)	1 (1.9)	3 (2.8)
Cevimeline hydrochloride	3 (5.5)	1 (1.9)	4 (3.7)
Pilocarpine	7 (12.7)	4 (7.4)	11 (10.1)
Pilocarpine hydrochloride	1 (1.8)	0	1 (0.9)
Cholinergic agonists (topical), n (%)	2 (3.6)	0	2 (1.8)
Pilocarpine	2 (3.6)	0	2 (1.8)
Antimalarial (systemic), n (%)	38 (69.1)	34 (63.0)	72 (66.1)
Chloroquine	0	2 (3.7)	2 (1.8)
Chloroquine phosphate	0	1 (1.9)	1 (0.9)
Hydroxycholoroquine	36 (65.5)	30 (55.6)	66 (60.6)
Hydroxycholoroquine sulfate	2 (3.6)	1 (1.9)	3 (2.8)
Glucocorticoids (local), n (%)	1 (1.8)	2 (3.7)	3 (2.8)
Clobetasol	0	2 (3.7)	2 (1.8)
Triamcinolone	1 (1.8)	0	1 (0.9)
cDMARD (local), n (%)	6 (10.9)	3 (5.6)	9 (8.3)
Ciclosporin	6 (10.9)	3 (5.6)	9 (8.3)

Data reported as n (%). cDMARD, conventional disease-modifying anti-rheumatic drug; DAZ, dazodalibep.

Table S3. List of Participating Institutional Review Boards and Ethics Committees

Country	IRB/EC	Name
Argentina	EC	Comité Institucional de Etica de Investigación en Salud (CIEIS-Hospital Privado - Universitario de Córdoba)- (Institutional Committee for Health Research Ethics)_ Córdoba
	EC	Comité de Ética de CER Investigaciones Clínicas CECIC
	EC	Comité Independiente de Ética para Ensayos en Farmacologia Clinica Fundacion de Estudios Farmacologicos y de Medicamentos 'Prof. Luis M. Zieher'
	EC	Comité Independiente de Ética para Ensayos en Farmacologia Clinica Fundacion de Estudios Farmacologicos y de Medicamentos 'Prof. Luis M. Zieher'
Mexico	EC	Comité de Ética en Investigación de la clínica de Investigación en Reumatología y Obesidad
	EC	Comité de ética en Investigación / Comité de Investigación de la Unidad Clínica de Bioequivalencia S. de R. L. de C.V.
	EC	Comité de Ética en Investigación de Investigación Biomédica para el desarrollo de fármacos / Comité de Investigación Biomédica para el Desarrollo de Fármacos
	EC	Comité de Ética en Investigación Del Hospital Hispano S.A de CV / Comité de Investigación del Hospital Hispano SA de CV
Peru	EC	Comité de Etica e Investigación del Hospital Cayetano Heredia (Committee of Ethics and Research of the Hospital Cayetano Heredia) Lima 31
	EC	Comité Institucional de Bioética (CIB) Via Libre_(Institutional Committee of Bioethics (CIB) Via Libre)_Lima
France	EC	CPP Ile-de-France 6 CPP IDF VI, Paris
Hungary	EC	Medical Research Council Ethics Committee for Clinical Pharmacology ETT KFEB
Italy	EC	COMITATO ETICO REGIONE TOSCANA - AREA VASTA NORD OVEST
Poland	EC	Dolnośląska Izba Lekarska Komisja Bioetyczna (Lower Silesian Chamber of Medicine, Bioethical Committee), Wrocław
United Kingdom	EC	North East – New Castle & North Tyneside 2 Research Ethics Committee - New Castle upon Tyne
United States	IRB	Copernicus Group Institutional Review Board
	IRB	Duke University Health Systems Institutional Review Board
	IRB	Tufts Health Sciences Institutional Review Board
	IRB	Johns Hopkins Medicine Institutional Review Board
Taiwan	IRB	Institutional Review Board of Chung Shan Medical University Hospital
	IRB	Institutional Review Board of Taichung Veterans General Hospital
	IRB	Chang Gung Medical Foundation Institutional Review Board
	EC	Research Ethics Committee, China Medical university Hospital
	IRB	Kaohsiung Veterans General Hospital Institutional Review Board
South Korea	IRB	The Catholic University of Korea Seoul St. Mary's Hospital Institutional Review Board
	IRB	Ajou University Hospital Institutional Review Board
	IRB	Gachon University Gil Medical Center Institutional Review Board
	IRB	Inha University Hospital Institutional Review Board
India	EC	Ethics Committee Sancheti Institute for Orthopedics & Rehabilitation, Pune
	EC	Institutional Ethics Committee, Chanre Rheumatology & Immunology Center & Research, Bangalore
	EC	Institutional Ethics Committee of Bangalore Medical College and Research Institute

	EC	Noble Hospital Institutional Ethics Committee (NHIEC),Pune
	EC	Institutional Ethics Committee-Cliniical Studies(IEC-CS), Apollo Hospitals, Bhubaneswar
	EC	KIMS Ethics Committee - Krishna Institute of Medical Sciences Limited, Secunderabad
	EC	Ethics Committee - Shalby Limited Shalby Hospital, Ahmedabad
	EC	Chennai Meenakshi Multispeciality Hospital Ethics Committee (CMMHEC), Mylapore Chennai

EC, ethics committee; IRB, Institutional Review Board

Figure S1. CXCL13 and RF through Crossover Period in Population #1 and Population #2

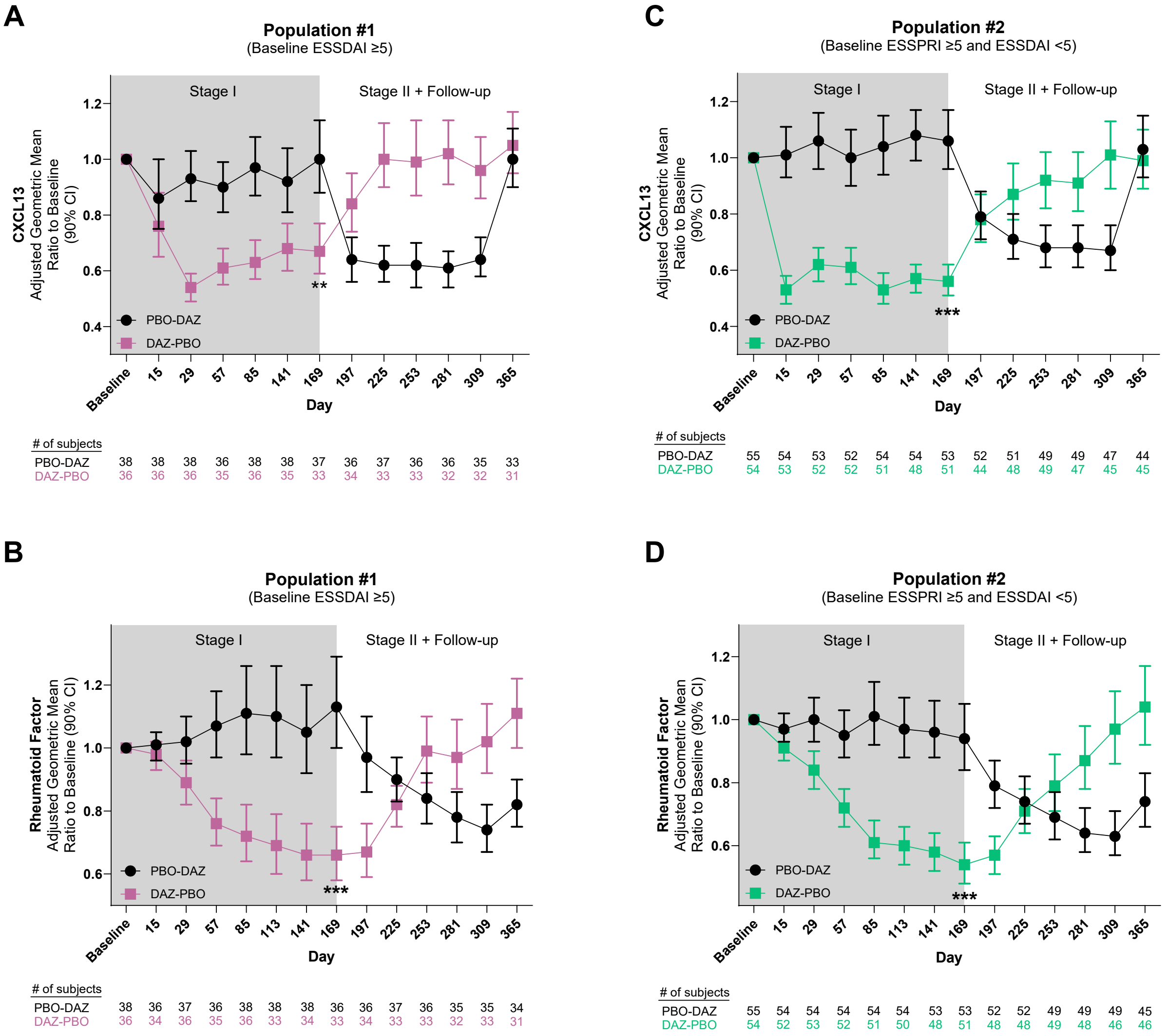

Data presented by study visit for Population #1 for (A) CXCL13 (analyzed by MMRM; Day 169 p = 0.0010; two-sided t-test), and (B) RF (analyzed by MMRM; Day 169 p < 0.0001; two-sided t-test). Data presented by study visit for Population #2 for (C) CXCL13 (analyzed by MMRM; Day 169 p < 0.0001; two-sided t-test), and (D) RF (analyzed by MMRM; Day 169 p < 0.0001; two-sided t-test). The PBO-DAZ group received PBO in Stage I and transitioned to DAZ in Stage II. The DAZ-PBO group received DAZ in Stage I and transitioned to PBO in Stage II. CI, confidence interval; DAZ, dazodalibep; ESSDAI, EULAR Sjögren's Syndrome Disease Activity Index; ESSPRI, EULAR Sjögren's Syndrome Patient Reported Index; ; MMRM, mixed-effect model for repeated measures; PBO, placebo; RF, rheumatoid factor; **p<0.01, ***p<0.001.
