# Supplementary material for: CD40 ligand antagonist dazodalibep in Sjögren’s disease: a randomized, double-blinded, placebo-controlled, phase 2 trial
Source: Nat Med. 2024 Jun 5;30(6):1583–92. doi: 10.1038/s41591-024-03009-3 (PMC11186761; doi:10.1038/s41591-024-03009-3)
Supplement: Supplementary file 2 — Reporting Summary [file 41591_2024_3009_MOESM2_ESM.pdf]

nature portfolio

Corresponding author(s): E. William St. Clair

Last updated by author(s): Apr 12, 2024

Reporting Summary

Nature Portfolio wishes to improve the reproducibility of the work that we publish. This form provides structure for consistency and transparency in reporting. For further information on Nature Portfolio policies, see our Editorial Policies and the Editorial Policy Checklist.

Statistics

For all statistical analyses, confirm that the following items are present in the figure legend, table legend, main text, or Methods section.

n/a	Confirmed	
☐	☒	The exact sample size (*n*) for each experimental group/condition, given as a discrete number and unit of measurement
☐	☒	A statement on whether measurements were taken from distinct samples or whether the same sample was measured repeatedly
☐	☒	The statistical test(s) used AND whether they are one- or two-sided<br>*Only common tests should be described solely by name; describe more complex techniques in the Methods section.*
☐	☒	A description of all covariates tested
☐	☒	A description of any assumptions or corrections, such as tests of normality and adjustment for multiple comparisons
☐	☒	A full description of the statistical parameters including central tendency (e.g. means) or other basic estimates (e.g. regression coefficient) AND variation (e.g. standard deviation) or associated estimates of uncertainty (e.g. confidence intervals)
☐	☒	For null hypothesis testing, the test statistic (e.g. *F*, *t*, *r*) with confidence intervals, effect sizes, degrees of freedom and *P* value noted<br>*Give P values as exact values whenever suitable.*
☒	☐	For Bayesian analysis, information on the choice of priors and Markov chain Monte Carlo settings
☒	☐	For hierarchical and complex designs, identification of the appropriate level for tests and full reporting of outcomes
☒	☐	Estimates of effect sizes (e.g. Cohen's *d*, Pearson's *r*), indicating how they were calculated

Our web collection on statistics for biologists contains articles on many of the points above.

Software and code

Policy information about availability of computer code

Data collection	No software was used.
Data analysis	Analysis was performed using SAS Version 9.4.

For manuscripts utilizing custom algorithms or software that are central to the research but not yet described in published literature, software must be made available to editors and reviewers. We strongly encourage code deposition in a community repository (e.g. GitHub). See the Nature Portfolio guidelines for submitting code & software for further information.

Data

Policy information about availability of data

All manuscripts must include a data availability statement. This statement should provide the following information, where applicable:

- Accession codes, unique identifiers, or web links for publicly available datasets
- A description of any restrictions on data availability
- For clinical datasets or third party data, please ensure that the statement adheres to our policy

Data sharing requests relating to data in this manuscript will be considered after the publication date and 1) this product and indication (or other new use) have been granted marketing authorization in both the US and Europe, or 2) clinical development discontinues and the data will not be submitted to regulatory authorities. There is no end date for eligibility to submit a data sharing request for these data.

This may include de-identified individual patient data for variables necessary to address the specific research question in an approved data-sharing request; also related data dictionaries, study protocol, statistical analysis plan, informed consent form, and/or clinical study report.

Qualified researchers may submit a request containing the research objectives, the Amgen product(s) and Amgen study/studies in scope, endpoints/outcomes of interest, statistical analysis plan, data requirements, publication plan, and qualifications of the researcher(s).

In general, Amgen does not grant external requests for individual patient data for the purpose of reevaluating safety and efficacy issues already addressed in the product labelling. A committee of internal advisors reviews requests. If not approved, requests may be further arbitrated by a Data Sharing Independent Review Panel. Requests that pose a potential conflict of interest or an actual or potential competitive risk may be declined at Amgen's sole discretion and without further arbitration.

Upon approval, information necessary to address the research question will be provided under the terms of a data sharing agreement. This may include anonymized individual patient data and/or available supporting documents, containing fragments of analysis code where provided in analysis specifications.

Further details are available at the following:
https://wwwext.amgen.com/science/clinical-trials/clinical-data-transparency-practices/clinical-trial-data-sharing-request

Research involving human participants, their data, or biological material

Policy information about studies with human participants or human data. See also policy information about sex, gender (identity/presentation), and sexual orientation and race, ethnicity and racism.

Reporting on sex and gender	The demographic table reports the breakdown of female and male participants in this study.
Reporting on race, ethnicity, or other socially relevant groupings	Participant demographics were self-reported. The demographic table reports the breakdown of race and ethnicity for participants in this study.
Population characteristics	Two populations of participants with Sjögren's disease were enrolled in this study. Population #1 consisted of participants with moderate-to-severe systemic disease activity (screening ESSDAI ≥5). Populations #2 consisted of participants with an unacceptable symptom burden and limited extraglandular systemic organ involvement (screening ESSPRI score ≥ 5 and screening ESSDAI score <5). Of the 139 participants screened for Population #1, 74 were randomized into the two treatment groups (dazodalibep: N=36; placebo: N=38). Of the 293 participants screened for Population #2, a total of 109 were randomized into the two treatment groups (dazodalibep: N=54; placebo: N=55)
Recruitment	This study recruited subjects globally at 63 sites. The small sample sizes in our trial may limit its generalizability as the study populations may not be representative of the entire population of patients with Sjögren's Disease.
Ethics oversight	All study participants provided written informed consent. This study was approved by appropriate Institutional Review Boards/Independent Ethics Committees (listed below):<br>1. Comité Institucional de Etica de Investigación en Salud (CIEIS-Hospital Privado - Universitario de Córdoba)- (Institutional Committee for Health Research Ethics)<br>2. Comité de Ética de CER Investigaciones Clínicas CECIC<br>3. Comité Independiente de Ética para Ensayos en Farmacologia Clinica Fundacion de Estudios Farmacologicos y de Medicamentos<br>4. Comité de Ética en Investigación de la clínica de Investigación en Reumatología y Obesidad<br>5. Comité de ética en Investigación / Comité de Investigación de la Unidad Clínica de Bioequivalencia S. de R. L. de C.V.<br>6. Comité de Ética en Investigación de Investigación Biomédica para el desarrollo de fármacos / Comité de Investigación Biomédica para el Desarrollo de Fármacos<br>7. Comité de Ética en Investigación Del Hospital Hispano S.A de CV / Comité de Investigación del Hospital Hispano SA de CV<br>8. Comité de Etica e Investigación del Hospital Cayetano Heredia (Committee of Ethics and Research of the Hospital Cayetano Heredia)<br>9. Comité Institucional de Bioética (CIB) Via Libre (Institutional Committee of Bioethics (CIB) Via Libre)<br>10. CPP Ile-de-France 6 CPP IDF VI, Paris<br>11.Medical Research Council Ethics Committee for Clinical Pharmacology ETT KFEB<br>12. COMITATO ETICO REGIONE TOSCANA - AREA VASTA NORD OVEST<br>13. Dolnośląska Izba Lekarska Komisja Bioetyczna (Lower Silesian Chamber of Medicine, Bioethical Committee), Wrocław<br>14. North East – New Castle & North Tyneside 2 Research Ethics Committee - New Castle upon Tyne<br>15. Copernicus Group Institutional Review Board<br>16. Duke University Health Systems Institutional Review Board<br>17. Tufts Health Sciences Institutional Review Board<br>18. Johns Hopkins Medicine Institutional Review Board<br>19. Institutional Review Board of Chung Shan Medical University Hospital<br>20. Institutional Review Board of Taichung Veterans General Hospital<br>21. Chang Gung Medical Foundation Institutional Review Board<br>22. Research Ethics Committee, China Medical university Hospital<br>23. Kaohsiung Veterans General Hospital Institutional Review Board<br>24. The Catholic University of Korea Seoul St. Mary's Hospital Institutional Review Board<br>25. Ajou University Hospital Institutional Review Board<br>26. Gachon University Gil Medical Center Institutional Review Board<br>27. Inha University Hospital Institutional Review Board<br>28. Ethics Committee Sancheti Institute for Orthopedics & Rehabilitation, Pune<br>29. Institutional Ethics Committee, Chanre Rheumatology & Immunology Center & Research, Bangalore

30. Institutional Ethics Committee of Bangalore Medical College and Research Institute
31. Noble Hospital Institutional Ethics Committee (NHIEC),Pune
32. Institutional Ethics Committee-Cliniical Studies(IEC-CS), Apollo Hospitals, Bhubaneswar
33. KIMS Ethics Committee - Krishna Institute of Medical Sciences Limited, Secunderabad
34. Ethics Committee - Shalby Limited Shalby Hospital, Ahmedabad
35. Chennai Meenakshi Multispeciality Hospital Ethics Committee (CMMHEC), Mylapore Chennai

Note that full information on the approval of the study protocol must also be provided in the manuscript.

Field-specific reporting

Please select the one below that is the best fit for your research. If you are not sure, read the appropriate sections before making your selection.

☒ Life sciences ☐ Behavioural & social sciences ☐ Ecological, evolutionary & environmental sciences

For a reference copy of the document with all sections, see nature.com/documents/nr-reporting-summary-flat.pdf

Life sciences study design

All studies must disclose on these points even when the disclosure is negative.

Sample size	The planned sample size of 72 participants in Population #1 (36 in the dazodalibep group and 36 in the placebo group) provided 80% power to detect a difference in mean change from baseline to Day 169 in ESSDAI of 3.0 (assumed standard deviation of 5) between the dazodalibep and placebo groups at a 2-sided alpha level of 0.10 using 2-sample t-test. The planned sample size of 102 participants in Population #2 (51 in the dazodalibep group and 51 in the placebo group) provided 80% power to detect a difference in mean change from baseline to Day 169 in ESSPRI of 1.0 (assumed standard deviation of 2) between the dazodalibep and placebo groups at a two-sided alpha level of 0.10 using 2-sample t-test.
Data exclusions	No data were excluded from the analysis.
Replication	This was the first clinical trial of dazodalibep in participants with Sjögren's Disease. Reproducibility of the results observed could not be tested but will be tested in future studies.
Randomization	Prior to randomization, participant eligibility must have been confirmed. Participants who met all eligibility criteria, including confirmation of eligibility, were randomized. A participant was considered randomized into the study when the Investigator notified the interactive voice/web response system (IXRS) that the participant met eligibility criteria and the IXRS provided the assignment of treatment group and allocated treatment, including study medication kit number. To ensure balanced rates of enrollment for the 2 populations, a procedure was implemented where enrollment of participants in Population #2 at each site was linked to enrollment of Population #1 participants at the site with a progressively increasing ratio of Population #2:Population #1 participants. At least one participant of the first 2 randomized at each site must have been from Population #1. Randomization was stratified by ESSDAI score at screening (< 10 points vs ≥ 10 points) for Population #1 and by ESSPRI score at screening (< 7.5 points vs ≥ 7.5 points) for Population #2.
Blinding	In this study, dazodalibep and the saline placebo were not identical in appearance. For maintaining the blinding of the participants, investigators, site staff, sponsor, contract research organization and staff, a local unblinded pharmacy staff member had the responsibility of allocating, dispensing and preparing the study medication, and covering the intravenous bags. A separate unblinded monitor was used for the oversight of study medication management. If treatment allocation for a participant became known to the investigator or other study staff involved in the management of study participants, the sponsor was notified immediately.

Reporting for specific materials, systems and methods

We require information from authors about some types of materials, experimental systems and methods used in many studies. Here, indicate whether each material, system or method listed is relevant to your study. If you are not sure if a list item applies to your research, read the appropriate section before selecting a response.

Materials & experimental systems

n/a	Involved in the study	
☒	☐	Antibodies
☒	☐	Eukaryotic cell lines
☒	☐	Palaeontology and archaeology
☒	☐	Animals and other organisms
☐	☒	Clinical data
☒	☐	Dual use research of concern
☒	☐	Plants

Methods

n/a	Involved in the study	
☒	☐	ChIP-seq
☒	☐	Flow cytometry
☒	☐	MRI-based neuroimaging

Clinical data

Policy information about clinical studies

All manuscripts should comply with the ICMJE guidelines for publication of clinical research and a completed CONSORT checklist must be included with all submissions.

Clinical trial registration: Clinicaltrials.gov: NCT04129164; EudraCT: 2019-002713-19

Study protocol: https://clinicaltrials.gov/study/NCT04129164

Data collection: This study was conducted from October of 2019 to March of 2023 at sites located worldwide: Argentina, Mexico, Peru, France, Hungary, Italy, Poland, United Kingdom, United States, Taiwan, South Korea, and India.

Outcomes: The primary endpoint for Population #1 was the change from baseline in ESSDAI at Day 169. Secondary endpoints for Population #1 included: the proportion of participants achieving ESSDAI [3] and ESSDAI [4] response (defined as a decrease of at least 3 or 4 points, respectively, from baseline in the ESSDAI at Day 169 without premature discontinuation from the study and without receiving rescue therapy), the change from baseline in FACIT-Fatigue score at Day 169, the change from baseline in OSDI at Day 169, and the change from baseline in PGIS at Day 169. The primary endpoint for Population #2 was the change from baseline in ESSPRI at Day 169. Secondary endpoints for Population #2 included the proportion of participants achieving an ESSPRI response (defined as ≥ 1 point or 15% reduction from baseline in ESSPRI score at Day 169 without premature discontinuation from the study and without receiving rescue therapy), the change from baseline in FACIT-Fatigue score at Day 169, the change from baseline in OSDI at Day 169, and the change from baseline in PGIS at Day 169.
